# Rapid characterization of the activities of lignin-modifying enzymes based on nanostructure-initiator mass spectrometry (NIMS)

**DOI:** 10.1186/s13068-018-1261-2

**Published:** 2018-09-27

**Authors:** Kai Deng, Jijiao Zeng, Gang Cheng, Jian Gao, Kenneth L. Sale, Blake A. Simmons, Anup K. Singh, Paul D. Adams, Trent R. Northen

**Affiliations:** 1 Joint BioEnergy Institute, Emeryville, CA 94608 USA; 2 Sandia National Laboratories, Livermore, CA 94551 USA; 3 Sichuan University of Science & Engineering, Zigong, 643000 Sichuan China; 4 Beijing University of Chemical Technology, Beijing, 100080 China; 5 Lawrence Berkeley National Laboratory, Berkeley, CA 94720 USA; 6 University of California, Berkeley, CA 94720 USA

**Keywords:** Lignin, β-Aryl ether, Lignin-modifying enzymes, NIMS, Enzyme assays

## Abstract

**Background:**

Producing valuable fuels and chemicals from lignin is a key factor for making lignocellulosic biomass economically feasible; however, significant roadblocks exist due to our lack of detailed understanding of how lignin is enzymatically depolymerized and of the range of possible lignin fragments that can be produced. Development of suitable enzymatic assays for characterization of putative lignin active enzymes is an important step towards improving our understanding of the catalytic activities of relevant enzymes. Previously, we have successfully built an assay platform based on glycan substrates containing a charged perfluorinated tag and nanostructure-initiator mass spectrometry to study carbohydrate active enzymes, especially various glycosyl hydrolyses. Here, we extend this approach to develop a reliable and rapid assay to study lignin-modifying enzymes.

**Results:**

Two β-aryl ether bond containing model lignin dimer substrates, designed to be suitable for studying the activities of lignin-modifying enzymes (LMEs) by nanostructure-initiator mass spectrometry (NIMS), were successful synthesized. Small-angle neutron scattering experiments showed that these substrates form micelles in solution. Two LMEs, laccase from the polypore mushroom *Trametes versicolor*, and manganese peroxidase (MnP) from white rot fungus *Nematoloma frowardii*, were tested for catalytic activity against the two model substrates. We show that the reaction of laccase and MnP with phenolic substrate yields products that arise from the cleavage of the carbon–carbon single bond between the α-carbon and the adjacent aryl carbon, consistent with the mechanism for producing phenoxy radical as reaction intermediates. Reactions of the nonphenolic substrate with laccase, on the other hand, adopt a different pathway by producing an α-oxidation product; as well as the cleavage of the β-aryl ether bond. No cleavage of the carbon–carbon bond between the α-carbon and the aryl carbon was observed. To facilitate understanding of reaction kinetics, the reaction time course for laccase activity on the phenolic substrate (I) was generated by the simultaneous measurement of all products at different time points of the reaction. Withdrawal of only a small sample aliquot (0.2 μL at each time point) ensured minimum perturbation of the reaction. The time course can help us to understand the enzyme kinetics.

**Conclusions:**

A new assay procedure has been developed for studying lignin-modifying enzymes by nanostructure-initiator mass spectrometry. Enzyme assays of a laccase and a MnP on phenolic and nonphenolic β-aryl ether substrates revealed different primary reaction pathways due to the availability of the phenoxy radical intermediates. Our assay provides a wealth of information on bond cleavage events not available using conventional colorimetric assays and can easily be carried out in microliter volumes and the quantitative analysis of product formation and kinetics is rapidly achieved by NIMS. This is the first time that NIMS technology was applied to study the activities of lignin-modifying enzymes. Unlike other previous works, our use of amphiphilic guaiacylglycerol β-*O*-4 substrate (I) enables the formation of micelles. This approach helps avoid the re-polymerization of the resulting monomeric product. As a result, our assay can clearly demonstrate the degradation pathways of phenolic guaiacylglycerol β-*O*-4 type of molecules with laccase and MnP.

**Electronic supplementary material:**

The online version of this article (10.1186/s13068-018-1261-2) contains supplementary material, which is available to authorized users.

## Background

Lignin is a major component of lignocellulosic biomass [1] and has the potential to be a valuable starting material for producing biofuels or high value bioproducts [2]. Moreover, conversion of lignin to fuels and chemicals [3–5] is regarded as a key factor for making lignocellulosic biomass economically feasible. The viability of lignin degradation depends on the availability of optimal lignin-modifying enzymes [6–9] that can efficiently degrade lignin biopolymers into simpler aromatics.

Enzymes responsible for lignin breakdown target the common lignin linkages [10, 11]: β-*O*-4 (arylglycerol-β-aryl ether), 5–5′ (biphenyl), β-5 (phenylcoumaran), 4-*O*-5 (diaryl ether), etc. Among these linkages, β-*O*-4 linkages represent approximately 50% and 60% of the total bonds of softwood and hardwood, respectively; making them important targets for enzyme assays. Hence, several surrogate substrates containing chromophores have been developed for studying cleavage of β-*O*-4 linkages. For example, guaiacylglycol and glycerol- β-*O*-(β-methylumbelliferyl) ethers have been synthesized for the fluorometric assays for β-etherases [12, 13]. As an alternative to spectroscopic assays, mass spectrometry-based enzyme activity assays enable detection of multiple products from a single substrate. One such approach uses nanostructure-initiator mass spectrometry (NIMS) [14–16] and glycan substrates [17] containing a charged perfluorinated tag, which partitions onto the NIMS surface, greatly enhancing detection of substrates and products.

Here, we describe the use of two synthetic β-aryl ether substrates for studying lignin-modifying enzymes using NIMS, a recently developed surface-based desorption ionization technique [14]. The NIMS chip is especially fabricated by etching the surface of a porous silicon wafer and treating it with an initiator molecule (e.g. a fluorous disiloxane). Samples are spotted onto the NIMS surface and then laser desorption/ionization of the analytes from the surface is used for the MS analysis. One interesting application of NIMS is called nimzyme [15] in which a fluorous tagged substrate (e.g., tagged glycans) is immobilized onto an NIMS surface. After incubating the surface with an *E. coli* lysate or a microbial community lysate, the NIMS surface was then washed. The products from the enzyme reaction can be detected by direct MS readout. More recently, nimzyme has been expanded to the use of soluble model substrates [16] to provide the specificity and sensitivity required to study glycosylhydrolases. One big advantage of the nimzyme method is multiplexing, which is achieved by simultaneous measurement of enzyme activities on multiple substrates. For multiplexing, different fluorous tags are typically needed for each substrate. The fluorous tag for each substrate is chosen to enable the differentiation of similar or identical products [17] from different enzymatic reactions by their unique mass tag (i.e., enabling different *m/z* in mass spectrometry). Nimzyme has been recently integrated with acoustic printing to create an enzyme characterization platform capable of one second/sample throughput, while only requiring only 20 μL sample volumes [18]. Currently, this platform has been used for measuring enzyme activities of glycosylhydrolases against a panel of well-characterized substrates [19].

Since approximately one-third of plant biomass is lignin, it is desirable to complement these existing assays for cellulose and hemicellulose active enzymes with assays designed to characterize lignin active enzymes. In addition, since NIMS is matrix free, data interpretation is readily facilitated, in comparison to MALDI-based assays that often use monolignols for desorption/ionization resulting in extensive lignin-related background ions.

Small-angle neutron scattering (SANS) reveals that these amphiphilic molecules form micelles like previous NIMS glycan substrates [16]. Micelles formation is important, and we hypothesize that the structure of micelles may reflect more natural reaction conditions in comparison to completely soluble substrates. Finally, the application of our LMEs assay demonstrates that phenolic and nonphenolic β-aryl ether substrates adopt different reaction pathways.

## Results and discussion

**Fig. 1:**
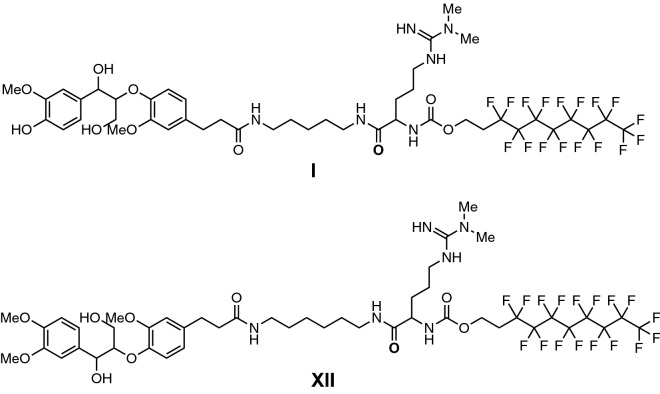
Structures of phenolic substrate I and nonphenolic substrate XII

**Fig. 2:**
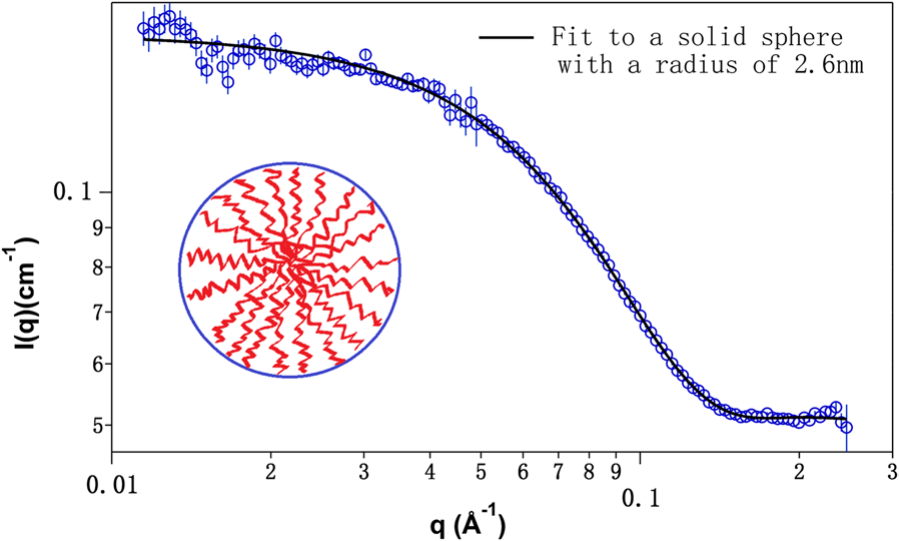
SANS data of 5 mg/mL phenolic β-aryl ether substrate (I) in D_2_O

Since the substrate molecule (Fig. 1) consists of a hydrophobic fluorinated segment and a hydrophilic arylglycerol, it is expected that the molecules will self-assemble in aqueous solutions as we have previously observed [16]. To examine this, small-angle neutron scattering (SANS) was performed with compound (**I**) in aqueous solution at 5 mM. The SANS data suggest the formation of micelles. As shown in Fig. 2, the SANS curve is fitted to a spherical model with a mean radius of 2.6 nm (polydispersity 11%). This dimension is consistent with the estimated size of these molecules, ~ 2.6 nm. A core–shell structure is not revealed by SANS data because the neutron scattering contrast between the hydrophobic and hydrophilic segments are similiar to each other [20]. These aggregates/micelles may reflect more natural reaction conditions in comparison to completely soluble substrate molecules often used in activity screening as many lignin-degrading enzymes act on insoluble solid lignin substrates.

**Fig. 3:**
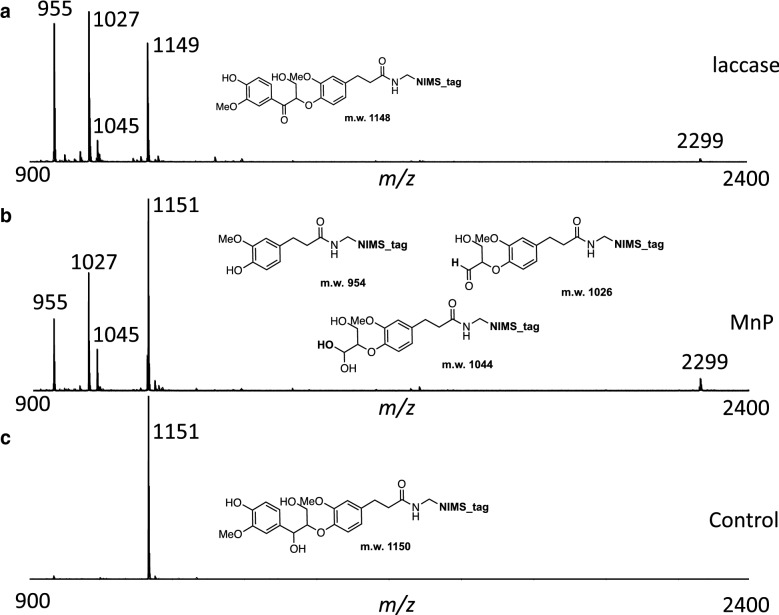
Representative mass spectral signatures from reactions of laccases and MnP with phenolic β-aryl ether substrate (**I**)

Figure 3 illustrates the results from the reactions of phenolic β-aryl ether dimeric substrates with two different lignin active enzymes, a laccase from the polypore mushroom *Trametes Versicolor*, and a manganese peroxidase (MnP) from the white rot fungus *Nematoloma frowardii*. Both reactions were performed at room temperature for 18 h. These results revealed that both enzymes generate similar product profiles, with slight differences in individual product concentration, suggesting that both enzymatic reactions may proceed via a similar mechanism.

It is interesting to see the formation of a small amount of product (*m/z* 2299) from the dimerization of phenolic β-*O*-4 substrate (*m/z* 1151), and no higher order of polymerization than dimerization was observed. Presumably, the micellar structure of the phenolic substrates forbids the access to the dimeric substrate from other phenoxy radicals. Over time, the alcohol in the dimeric product (*m/z* 2299) was found to be oxidized by enzymes to the corresponding carbonyl products as shown in the mass spectra (peak with *m/z* 2297). It seems that only one hydroxyl group is being oxidized, because no peaks corresponding to the products with multiple oxidation were observed.

The formation of this dimeric product most likely occurred through a phenoxy radical intermediate generated by the oxidation of the phenol subunit. Dimerization of the phenolic β-*O*-4 substrate via intermolecular phenoxy radical cross-coupling would produce product with the construction of carbon–carbon or carbon–oxygen bonds like the 5–5 or 4-*O*-5 linkages in lignin [21]. According to the NMR studies by Butler et al. [21], the product from dimerization is very likely to be formed by the 5–5 linkage.

Previously, Rittstieg et al. [22] used guaiacylglycerol-β-guaiacyl ether, a compound with structure similar to substrate (I) but without the NIMS tag to study laccase activity. In their study, a polymeric precipitate was observed following treatment of the substrate with laccase, which was attributed to a free-radical initiated polymerization reaction. Interestingly, we did not observe any precipitation in the present study. We attribute this to a putative influence of the NIMS probe, which may inhibit polymerization, meaning that only monomeric compounds are formed. This finding is consistent with the work of Gold et al. [23] who added a methoxy group at the 5′ position to prevent polymerization. While indeed the NIMS probe may be advantageous for the prevention of polymerization, it may also introduce bias due to the interactions of the fluorous tag with the enzyme and this topic will be an important area for future investigation.

**Fig. 4:**
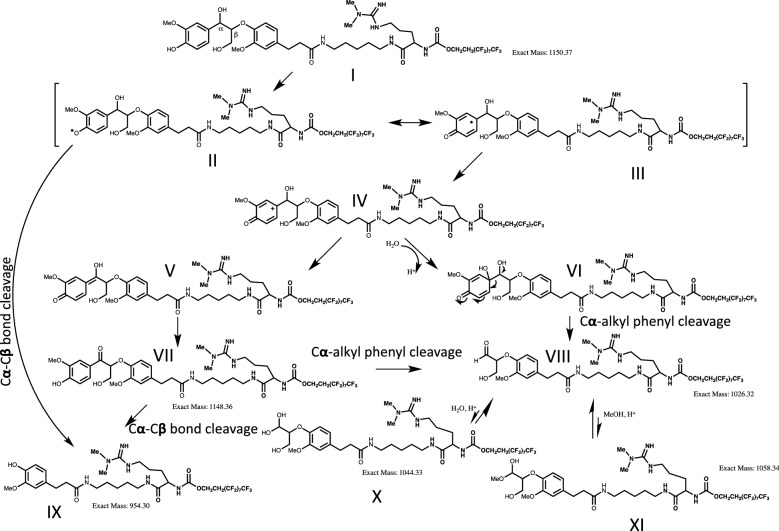
Possible mechanism of product formation in enzymatic reaction of phenolic β-aryl ether substrate (**I**)

The potential mechanism for the formation of products from phenolic β-aryl ether substrate (I) is shown in Fig. 4. The phenoxy radical intermediate II, generated by the oxidation of phenolic substrate I by laccase through single electron transfer, can delocalize to form resonance structure III. The loss of another electron from this carbon radical by laccase oxidation affords an important cation intermediate IV. Two distinct pathways are probably operational here and depend on the phenoxy radical intermediate (II). Pathway 1 involves the C_**α**_–C_**β**_ bond cleavage [23–25], followed by rapid cleavage of the C_**β**_–*O* ether bond to form product IX. C_**α**_–C_**β**_ bond cleavage can also proceed through the phenoxy radical intermediates of the phenolic β-aryl ether substrate **I** or its C_α_-oxo-product VII. Alternatively, the carbon–carbon single bond between the C_**α**_ and the aryl carbon can be cleaved. Again, the phenoxy radical is the intermediate needed to initiate the entire reaction cascade to produce aldehyde product VIII. For both reactions of laccase and MnP with phenolic β-aryl ether substrate, aldehyde product VIII is consistently produced in higher amounts than product IX, which indicates that the pathway with the cleavage of the C_α_-alkyl phenyl bond is predominant relative to the pathway with the C_α_–C_β_ bond cleavage. Product X (with *m/z* 1045) is presumably the hydrate form of aldehyde VIII.

Interestingly, when methanol was added to quench the reaction, the peak corresponding to the hydrate of aldehyde (*m/z* 1045) was reduced significantly and the new peak appearing at *m/z* 1059 was consistent with the formation of a product from the methanolysis of aldehyde VIII. When deuterated methanol (CD_3_OD) was used to quench the reaction, as expected, the mass peak with *m/z* 1062 appeared. These results confirmed the identity of aldehyde VIII.

**Fig. 5:**
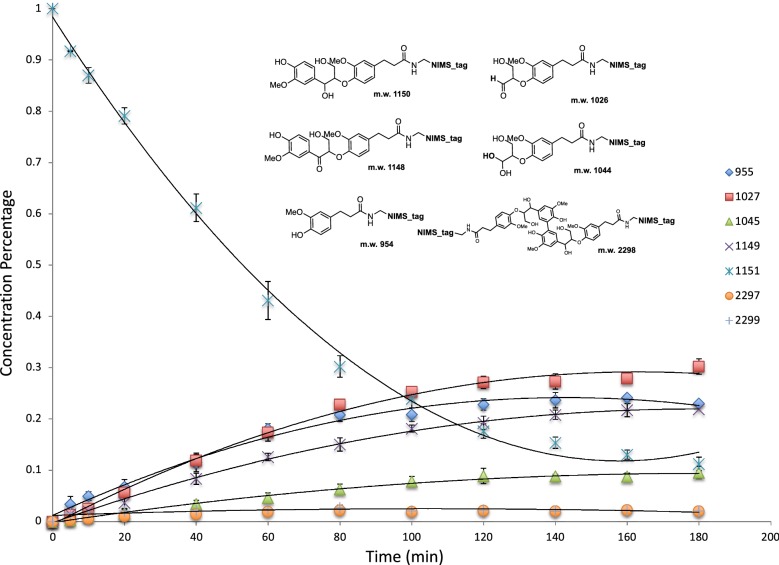
Time course of laccases with phenolic β-aryl ether substrate (**I**) over 3 h time period: 955 (**IX** in Fig. 4, green diamond); 1027 (**VIII**, red square); 1045 (**X**, green up triangle); 1149 (**IV**, purple cross); 1151 (**I**, pale blue asterisk); 2297 (orange solid circle); 2299 (gray plus sign)

The time course of an enzymatic reaction contains valuable information about the properties of the enzyme. By obtaining product distribution at different time points in the enzymatic reaction, we can determine the reaction pathways, bottleneck steps in the reaction and key enzyme parameters, etc. In our NIMS-based enzyme assay, substrate and products concentrations were measured by the relative intensity of the *m/z* signals of each compound. It is important to point out a few attributes of these assays. Since both substrates and products possess highly similar fluorous tails and, thus, the dimethyl arginine groups should enable different molecules with similar ionization efficiency in NIMS, so it is possible to directly compare ion intensities as an approximation of substrate and product concentrations. Also, since multiple products are measured simultaneously from a 0.2 μL sample (from a 10 μL reaction) at each time point the disturbance of the reaction is limited. These analyses also consume little of the precious synthetic substrates. Figure 5 shows the reaction time course of laccase with phenolic β-aryl ether substrate (I) at room temperature over a time period of 180 min. Over the course of the experiment, a continuous decrease of the phenolic β-aryl ether substrate is observed along with an increase in the product formation (products IX, VII, VIII) with the exception of the dimerization product which, after initial increase in relative abundance in the first h, was completely converted to oxidation product (with *m/z* 2297).

**Fig. 6:**
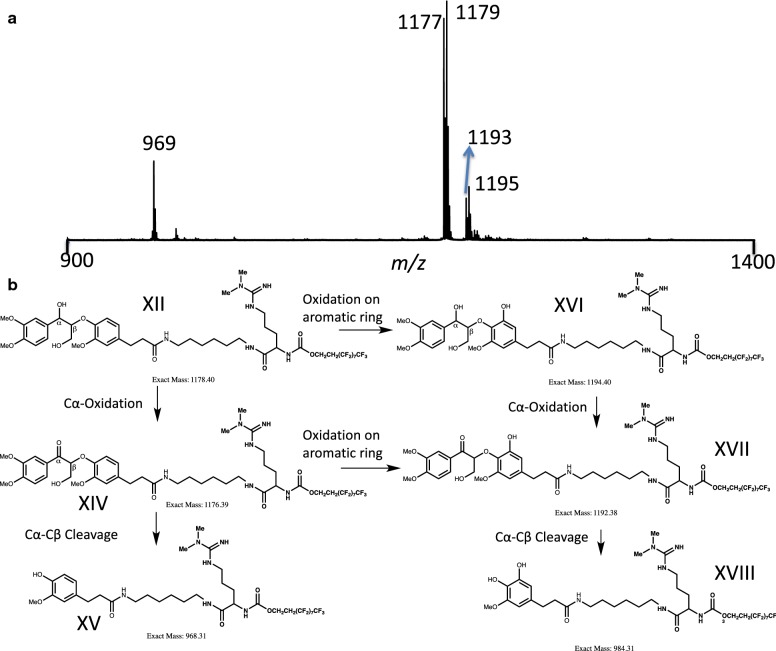
Mass spectra (**a**) and possible mechanism (**b**) of laccases with nonphenolic β-aryl ether substrate **XII**. (1) The exact position of the additional hydroxyl group on the aromatic ring is uncertain. For simplicity, the drawing shows one of the three possible positions. (2) No activity was detected for MnP against the nonphenolic substrate

As for the reaction of laccase with nonphenolic β-aryl ether substrate (Fig. 6), temperatures of 37 °C and longer reaction times were needed to accurately measure reaction products, and products profiles are different from those obtained when using the phenolic β-aryl ether substrate. Since there is no phenoxy radical intermediate, no product from the cleavage of carbon–carbon single bond between the C_α_ and the adjacent aryl carbon was observed. C_α_-oxo-product XIV is predominant compared to product XV, which comes from the C_α_–C_β_ bond cleavage. In addition, HOBt was added to the reaction as a mediator compared to no HOBt being added to the reaction of laccase with phenolic substrate (I). It is known that laccase reactivity decreases with an increase of the steric encumbrance; the use of mediator, HOBt, can help to overcome the problems related to substrate accessibility [9]. In this case, the nonphenolic substrate (XII) may have greater difficulty entering the laccase active site compared to phenolic substrate (I). HOBt is a small molecule that can be oxidized by laccase to an intermediate with high redox potential, which can oxidize the nonphenolic substrate (XII) to the corresponding products. More interestingly, some products, likely from the oxidation of the phenyl ring [26], were observed as evidenced by *m/z* 1193, 1195, which correspond to the addition of a hydroxyl group on the aromatic ring [26] of substrates XII and C_α_-oxo-product XIV. The location of the hydroxyl group on the ring is still unclear; however, a small peak with *m/z* 985 was detected which may indicate that the oxidation occurs on the phenyl ring linked to the C_**β**_ through oxygen. When using identical reaction conditions, but without HOBt, only the substrate was detected using mass spectrometry.

The structure of our model compounds is designed by coupling a fluorous tag with the β-*O*-4 dimer through a carbon chain. This design offers the opportunity to construct enantiomeric pure substrates with ease. The β-*O*-4 dimer itself has two chiral centers; therefore, there are four possible enantiomers. It is known that enzymes are chiral, and they can distinguish among enantiomers [27, 28]. To understand enzyme specificity, enantiomeric pure substrates are needed. Our approach (shown here) could easily be extended to prepare these important chiral substrates (e.g., each with different mass tags attached to ensure unique identity in mass spectrometry) by synthetic organic chemistry to study the enzyme specificity. Moreover, due to the multiplexing nature of our NIMS assays, multi-substrate/product systems can be utilized to thoroughly investigate the enzymatic activities/specificities. Progress in this area will be reported in the future. In addition to the approach using model compounds for the study of LMEs, development of post-reaction derivatization approach like those we have reported for analysis of glycosyl hydrolases [29] is an important direction for future research. These studies would have the advantages of minimizing experimental bias and allow analysis of enzymes against the most process relevant feedstocks.

## Conclusions

In summary, we have synthesized two β-aryl ether substrates suitable for high-throughput assaying of lignin-modifying enzymes by NIMS. In solution, these amphiphilic molecules tend to form micelles. Use of these substrates to assay a laccase and a manganese peroxidase revealed that phenolic and nonphenolic β-aryl ether substrates demonstrate different primary reaction pathways due to the availability of the phenoxy radical intermediates. Further work will focus on preparing other model lignin compounds suitable for NIMS analysis to study catalysis of breaking lignin bond linkages. Together with the substrates already prepared for studying the activities of cellulases and hemicellulases, this pool of substrates forms the basis for a powerful mass spectrometry-based multiplexing assay, which has the ability to simultaneously detect multiple functions of enzymes (or enzyme cocktails) responsible for the deconstruction of lignocellulosic biomass. Ultimately, this approach may potentially aid in identifying more efficient, low cost enzyme cocktails useful for converting all biomass polymers into valuable bioproducts.

## Experimental section

### Synthesis

The syntheses of phenolic and nonphenolic β-*O*-4 aryl ether model compounds are outlined in Additional file 1: Scheme S1 and S2 and detailed synthetic procedures are provided in Additional file 1.

### Enzymes

The laccases (0.5 U/mg, solid cultures from *Trametes versicolor*) and manganese peroxidases (solid cultures from the white rot fungus, *Nematoloma frowardii*, Cat. No. EN-201S) used in this study were purchased from Aldrich and Jena Bioscience, respectively.

### Enzyme assays and conversion of products

A. Laccase and manganese peroxidase (MnP) with phenolic substrate **I**.
  1. Laccase reaction. Prepare laccase (solid, 0.5 U/mg from Aldrich) stock solution with a concentration of 1.5 mg/mL in sodium acetate buffer (100 mM, pH 4.6). Then, to a clear, thick-walled 0.2 mL PCR tube (Axygen) add 10 μL of the above laccase solution and 1 μL of phenolic substrate **I** (e.g., 5 mM stock solution in D.I. water). The resulting mixture was mixed by a Vortex mixer and incubated at room temperature with indicated time.
  2. MnP reaction. Prepare MnP (solid, 200 U/mg from Jena Bioscience) stock solution with a concentration of 1.0 mg/mL in sodium acetate buffer (100 mM, pH 4.6). Next prepare the experimental buffer solution by adding solid sodium malonate and MnCl_2_ to sodium acetate buffer (100 mM, pH 4.6) to make the final concentration of sodium malonate and MnCl_2_ as 50 mM and 10 mM, respectively. Then, to a clear, thick-walled 0.2 mL PCR tube (Axygen) add 5 μL of the above MnP stock solution, 5 μL of the above experimental buffer solution (containing sodium malonate and MnCl_2_), and one μL of phenolic substrate **I** (5 mM stock solution in D.I. water). The resulting mixture was mixed by a Vortex mixer and incubated at room temperature with indicated time.
B. Laccase with nonphenolic substrate **XII**.
  Prepare laccase (solid, 0.5 U/mg from Aldrich) stock solution with a concentration of 1.5 mg/mL in sodium acetate buffer (100 mM, pH 4.6). Then, to a clear, thick-walled 0.2 mL PCR tube (Axygen) add 10 μL of the above laccase solution, one μL of phenolic substrate **XII** (5 mM stock solution in D.I. water) and 1 μL of 1-hydroxybenzotriazole (HOBt, 3.4 mM stock solution in D.I. water). The resulting mixture was mixed by a Vortex mixer and incubated at 37 °C with indicated time.

### Nanostructure-initiator mass spectrometry (NIMS)

In each case, 0.2 µL of the quenched reaction sample was spotted onto the NIMS surface and removed after 30 s. A grid drawn manually on the NIMS chip using a diamond-tip scribe helped with spotting and identification of sample spots in the spectrometer. Chips were loaded using a modified standard MALDI plate. NIMS analysis was performed using a 4800 MALDI TOF/TOF mass analyzer from Applied Biosystems (Foster City, CA). Signal intensities were identified for the ions of the products and ~ 1000 laser shots were collected.

## Additional file

**Additional file 1.** The synthesis and characterization of Model substrates.

## Abbreviations

NIMS: nanostructure-initiator mass spectrometry
LMEs: lignin-modifying enzymes
MnP: manganese peroxidases
MALDI-TOF: matrix-assisted laser desorption ionization-time of flight mass spectrometry
CD_3_OD: deuterated methanol
SANS: small-angle neutron scattering
D.I. water: deionized water
PCR tube: polymerase chain reaction tube
MnCl_2_: manganese(II) dichloride
