# Supplementary material for: Rapid characterization of the activities of lignin-modifying enzymes based on nanostructure-initiator mass spectrometry (NIMS)
Source: Biotechnol Biofuels. 2018 Sep 27;11:266. doi: 10.1186/s13068-018-1261-2 (PMC6158898; doi:10.1186/s13068-018-1261-2)
Supplement: Supplementary file 1 — Additional file 1. The synthesis and characterization of Model substrates. [file 13068_2018_1261_MOESM1_ESM.pdf]

Additional File 1: The synthesis and characterization of model compounds

(Experimental procedures, spectroscopic data, and 1H and 13C NMR spectra)

Rapid Characterization of the Activities of Lignin-Modifying Enzymes Based on Nanostructure-Initiator Mass Spectrometry (NIMS)

Kai Deng*,[a,b] Jijiao Zeng [a,b], Gang Cheng[c], Jian Gao [d], Kenneth Sale, [a,b] Blake A. Simmons,[a,d] Anup K. Singh,[a,b] Paul D. Adams [a,d,e] and Trent R. Northen*[a,d]

[a]Joint BioEnergy Institute, Emeryville, CA 94608

[b]Sandia National Laboratories, Livermore, CA 94551

[c]Beijing University of Chemical Technology, Beijing, China 100080

[d]Lawrence Berkeley National Laboratory, Berkeley, CA 94720

[e]University of California, Berkeley, CA 94720

Table of Contents

General Information……………………………………………………………………page S2
Reaction Schemes and Experimental Procedure for Compounds…………… page S3
Spectra for Compounds…………………………………………………………………page S14

General Information about Organic Synthesis

All chemicals were purchased as reagent grade and used without further purification. Flash column chromatography steps were performed on a CombiFlash Rf chromatography system from Teledyne ISCO (Lincoln, NE). Reactions were monitored using analytical thin-layer chromatography (TLC) in EM silica gel 60 F254 plates and developed by acidic ceric ammonium molybdate or potassium permanganate TLC stains. ^{1}H NMR and ^{13}C NMR spectra were recorded on either a Bruker AV-600 or AVB-400. Chemical shifts (in ppm) were assigned according to the internal standard signal of $CDCl_3$ (δ = 7.26 ppm), CD_3OD (δ = 3.31 ppm), or $CDCl_3$ (δ = 77.16 ppm) and CD_3OD (δ = 49.00 ppm) for ^{13}C NMR. Coupling constants (J) are reported in Hertz, and the splitting patterns are described by using the following abbreviations: s, singlet; d, doublet; dd, doublet of doublets; ddd, doublet of doublet of doublets; t, triplet; dt, doublet of triplets; q, quartet; m, multiple; AB, AB spin system. High-resolution mass spectral data were obtained from the University of California, Berkeley Mass Spectral Facility. Laccases (0.5 U/mg) and Manganese peroxidases (cat. No. EN-201S) used in this study was purchased from Aldrich and Jena Bioscience, respectively.

Synthesis of Phenolic β-O-4 substrate S11

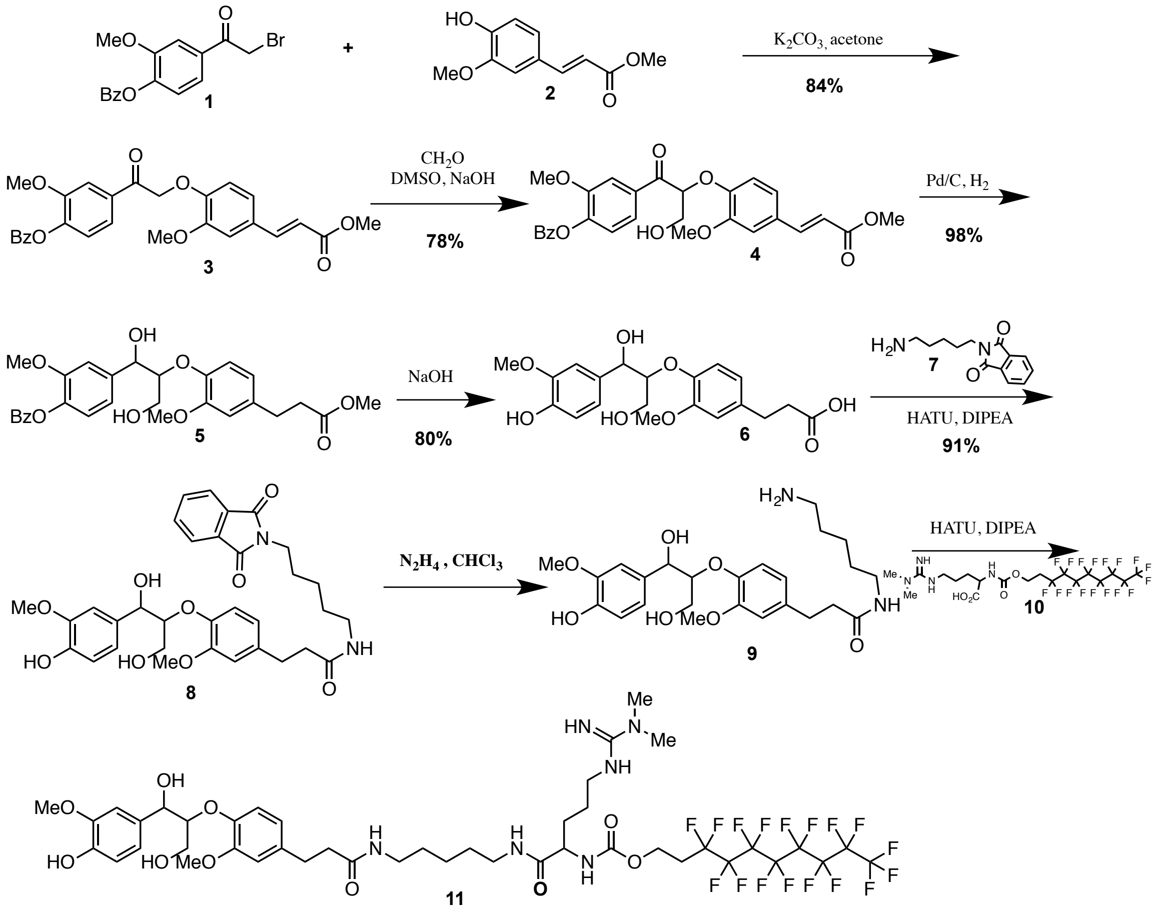

62% over two steps

Scheme 1 Synthesis of phenolic β-aryl ether substrate 11

As shown in Scheme 1, synthesis of the phenolic β-O-4 substrate started with alkylation of a known bromide 4-(2-bromoacetyl)-2-methoxyphenyl benzoate (1) with methyl (E)-3-(4-hydroxy-3-methoxyphenyl)acrylate. (2)(3) With compound 3 in hand, an aldol condensation with formaldehyde produced compound 4 in 78% yields. (4) Hydrogenation with Pd/C as the catalyst under 1 atmosphere hydrogen not only reduces the carbon-carbon double bond of the α,β-unsaturated methyl ester, but also reduced the Cα-ketone to the corresponding secondary alcohol. Presumably the adjacent phenyl ring facilitates the reduction of the ketone. Saponication (5) of the methyl ester by treating compound 5 with sodium hydroxide provided the desired carboxylic acid 6, with the protecting group of benzoyl on phenol being removed as well. The acid 6 and amine 7 are linked by HATU (1-[Bis(dimethylamino)methylene]-1H-1,2,3-triazolo[4,5-b]pyridinium 3-oxid hexafluorophosphate) mediated amide bond formation (6) to afford compound 8. The phthalimide protection group was removed by hydrazine. (7) Another HATU

mediated amide formation between amine 9 an acid 10 afforded the desired phenolic β-O-4 substrate 11.

Detailed Reaction Steps Leading to Compound 11

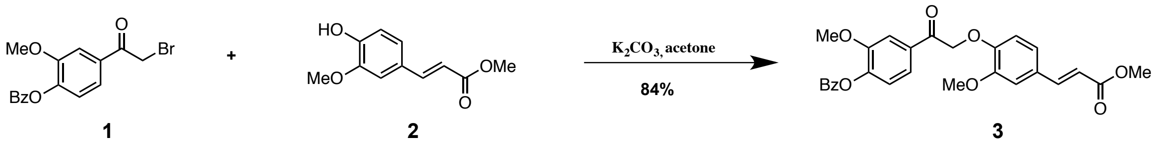

(*E*)-2-Methoxy-4-(2-(2-methoxy-4-(3-methoxy-3-oxoprop-1-en-1-yl)phenoxy)acetyl)phenyl benzoate (3)
To a stirred solution of 4-(2-bromoacetyl)-2-methoxyphenyl benzoate (6) 1 (4.48 g, 12.8 mmol) and Methyl 3-(4-OH-3-OMe)cinnamate **2** (3.20 g, 15.4 mmol) in acetone (100 mL) was added K_2CO_3 (2.00 g, 7.62 mmol).(2) The resulting mixture was stirred at 52 °C for 3.5 h and the solvent was evaporated under reduced pressure and the resulting residue was redissolved in ethyl acetate. After filtration and concentration under reduced pressure, the residue was subjected to column purification to give 5.12 g **compound 3** in 84% yield. ^{1}H NMR (600 MHz, $CDCl_3$) δ (ppm) 8.21-8.17 (dd, 2H, *J* = 9.6, 1.2 Hz), 7.68-7.66 (d, 1H, *J* = 1.8 Hz), 7.65-7.61 (dt, 2H, *J* = 8.4, 1.8 Hz), 7.61-7.57 (d, 1H, *J* = 15.6 Hz), 7.52-7.48 (t, 2H, *J* = 8.4 Hz), 7.27-7.24 (d, 1H, *J* = 8.4 Hz), 7.07-7.04 (d, 1H, *J* = 1.8 Hz), 7.03-6.99 (dd, 1H, *J* = 8.4, 1.8 Hz), 6.80-6.77 (d, 1H, *J* = 8.4 Hz), 6.32-6.27 (d, 1H, *J* = 15.6 Hz), 5.39-5.34 (s, 2H), 3.89 (s, 3H), 3.84 (s, 3H), 3.77 (s, 3H). ^{13}C NMR (150 MHz, $CDCl_3$) 192.8, 167.4, 164.1, 151.9, 149.7, 149.4, 144.7, 144.4, 133.8, 133.1, 130.3, 128.8, 128.7, 128.6, 123.2, 122.1, 121.3, 116.1, 113.9, 111.8, 110.7, 71.6, 56.1, 55.9, 51.5. HRMS (ESI) *m/z*: Calcd for $C_{27}H_{24}O_8Na$ ($M+Na^+$) 499.1363, found 499.1359.

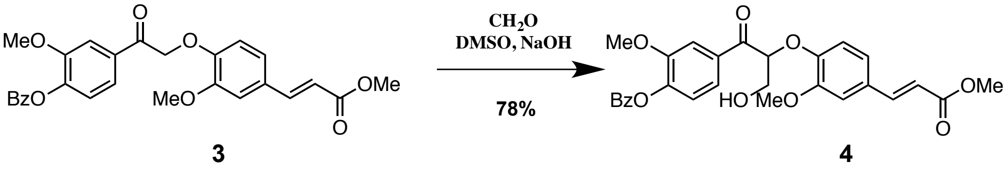

(*E*)-4-(3-hydroxy-2-(2-methoxy-4-(3-methoxy-3-oxoprop-1-en-1-yl)phenoxy)propanoyl)-2-methoxyphenyl benzoate (4)
To a stirred solution of **compound 3** (700 mg, 1.47 mmol) in DMSO (4 mL) was added formaldehyde power (50 mg, 1.67 mmol) and K_2CO_3 (61 mg, 0.43 mmol). The resulting mixture was stirred at room temperature for 2 h. The resulting residue was subjected to column purification to give 800 mg **compound 4** in 78% yield. ^{1}H NMR (600 MHz, $CDCl_3$) δ (ppm) 8.18-8.15 (dd, 2H, *J* = 7.8, 1.8 Hz), 7.75-7.72 (dd, 1H, *J* = 8.4, 1.8 Hz), 7.70-7.68 (d, 1H, *J* = 1.8 Hz), 7.63-7.59 (dt, 1H, *J* = 7.8, 2.4 Hz), 7.57-

7.52 (d, 1H, J = 15.6 Hz), 7.50-7.45 (t, 2H, J = 8.4 Hz), 7.24-7.21 (d, 1H, J = 8.4 Hz), 7.01-7.00 (d, 1H, J = 1.8 Hz), 6.97-6.93 (dd, 1H, J = 8.4, 1.8 Hz), 6.78-6.75 (d, 1H, J = 8.4 Hz), 6.28 -6.24 (d, 1H, J = 15.6 Hz), 5.59-5.56 (dd, 1H, J = 6.0, 3.6 Hz), 4.58-4.10 (m, 2H), 3.81 (s, 3H), 3.80 (s, 3H), 3.74 (s, 3H). ^{13}C NMR (150 MHz, $CDCl_3$) 194.9, 167.4, 164.1, 151.8, 149.8, 148.6, 144.7, 144.3, 133.8, 133.4, 130.3, 129.2, 128.8, 128.6, 123.2, 122.2, 122.0, 116.4, 116.0, 112.4, 110.9, 83.3, 63.4, 56.0, 55.8, 51.6. HRMS (ESI) *m/z*: Calcd for $C_{28}H_{26}O_9Na$ (M+Na$^+$) 529.1469, found 529.1463.

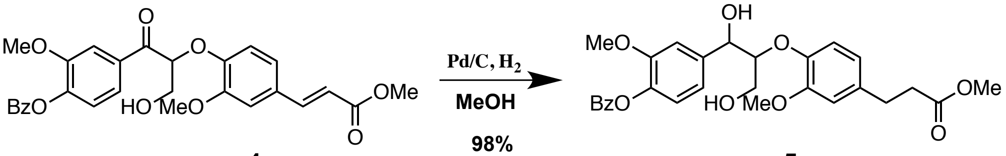

4 5

4-(1,3-dihydroxy-2-(2-methoxy-4-(3-methoxy-3-oxopropyl)phenoxy)propyl)-2-methoxyphenyl benzoate (5)

To a stirred solution of **compound 4** (450 mg, 0.89 mmol) in methanol (25 mL) was added Pd/C (100 mg, 0.047 mmol). The system was purged with hydrogen three times before stirring under 1atm hydrogen gas at room temperature for 15 h. After filtration and concentration under reduced pressure, the resulting residue was subjected to column purification to give 445 mg **compound 5** as two diastereomers with ratio of 7:3 in 98% total yield. ^{1}H NMR (600 MHz, $CDCl_3$) δ (ppm) 8.24-8.20 (m, 2H), 7.66-7.61 (m, 2H), 7.54 (m, 2H), 7.17-7.15 (d, 0.7H, J = 1.8 Hz), 7.15-7.13 (m, 1H), 7.13-7.11 (d, 0.3H, J = 1.8 Hz), 7.09-7.05 (dd, 0.7H, J = 7.2, 1.2 Hz), 7.03-7.00 (d, 0.7H, J = 7.2 Hz), 7.00-6.98 (dd, 0.3H, J = 6.6, 1.2 Hz), 5.08-5.05 (dd, 0.7H, J = 6.6, 1.8 Hz), 5.04-5.02 (dd, 0.3H, J = 7.2, 1.2 Hz), 4.19-4.16 (m, 0.3H), 4.03-4.01 (m, 0.7H), 3.96-3.91(m, 0.3H), 3.88 (s, 2.1H), 3.85 (s, 0.9H), 3.81 (s, 2.1H), 3.80 (s, 0.9H), 3.680 (s, 2.1H), 3.678 (s, 0.9H), 3.58 (m, 0.7H), 3.20-3.16 (t, 0.3H, J = 5.4 Hz), 3.15-3.09 (t, 0.7H, J = 5.4 Hz), 2.95-2.89 (m, 2H), 2.633 (t, 1.4H, J = 6.6 Hz), 2.630 (t, 0.6H, J = 6.6 Hz), 2.921 (t, 1.4H, J = 6.6 Hz), 2.919 (t, 0.6H, J = 6.6 Hz).^{13}C NMR (150 MHz, $CDCl_3$) Major diastereomer: 173.31, 164.723, 151.36, 150.96, 145.89, 139.61, 138.96, 136.67, 133.47, 130.29, 129.36, 128.54, 122.77, 121.14, 120.75, 119.40, 112.32, 111.14, 88.83, 73.67, 61.02, 55.99, 55.88, 51.70, 35.74, 30.68; Minor diastereomer: 173.32, 164.768, 151.30, 151.20, 145.26, 139.50, 139.21, 136.57, 133.47, 130.29, 129.36, 128.54, 122.67, 121.09, 120.60, 118.50, 112.35, 110.53, 86.89, 72.79, 60.83, 55.98, 55.88, 51.70, 35.74, 30.68
HRMS (ESI) *m/z*: Calcd for $C_{28}H_{30}O_9Na$ (M+Na$^+$) 533.1782, found 533.1773.

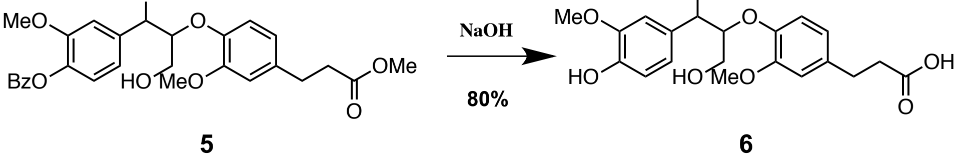

5 6

3-(4-((1,3-dihydroxy-1-(4-hydroxy-3-methoxyphenyl)propan-2-yl)oxy)-3-methoxyphenyl)propanoic acid (6)

To a stirred solution of **compound 5** (330 mg, 0.65 mmol) in a mixture of methanol (4 mL) and water (2 mL) was added NaOH (2.6 mL, 0.5 M). The resulting mixture was stirred at room temperature for 2 h. After quenching the reaction with HCl (5 mL, 0.5 M), Ethyl acetate was used for extraction. The combined organic layers were concentrated under reduced pressure and the resulting residue was subjected to column purification to give 200 mg **compound 6** as two diastereomers with ratio of 7:3 in 80% total yield. ^{1}H NMR (600 MHz, $CDCl_3$) δ (ppm) 7.03-7.00 (d, 0.7H, *J* = 8.4 Hz), 6.97-6.94 (m, 1H), 6.92-6.89 (dd, 0.7H, *J* = 7.8, 1.8 Hz), 6.88-6.87 (m, 1H), 6.845-6.825 (d, 0.3H, *J* = 7.8 Hz), 6.82-6.80 (dd, 0.3H, *J* = 7.8, 1.8 Hz), 6.79-6.77 (d, 0.7H, *J* = 1.2 Hz), 6.77-6.75 (dd, 0.7H, *J* = 5.4, 1.8 Hz), 6.75-6.74 (d, 0.3H, *J* = 1.2 Hz), 6.74-6.71 (dd, 0.3H, *J* = 8.4, 1.8 Hz), 4.05-4.01 (m, 0.3H), 4.00-3.96 (m, 0.7H), 3.872 (s, 2.1H), 3.864 (s, 2.1H), 3.858 (s, 0.9H), 3.840 (s, 0.9H), 3.68-3.64 (dd, 0.3H, *J* = 12.6, 3.0 Hz), 3.63-3.59 (dd, 0.7H, *J* = 12.6, 3.0 Hz), 3.49-3.44 (m, 1H), 2.93-2.86 (m, 3H), 2.66-2.58 (m, 3H).^{13}C NMR (150 MHz, $CDCl_3$) Major diastereomer: 176.60, 150.99, 146.64, 145.95, 145.53, 136.59, 131.45, 121.12, 120.88, 120.18, 114.31, 112.32, 109.41, 89.36, 73.93, 60.74, 55.94, 55.82, 35.49, 30.43; Minor diastereomer: 176.63, 150.99, 146.59, 145.95, 145.53, 136.49, 131.85, 121.06, 120.75, 120.13, 114.23, 112.32, 109.41, 87.21, 72.72, 61.67, 55.85, 55.73, 35.49, 30.43.
HRMS (ESI) *m/z*: Calcd for $C_{20}H_{24}O_8Na$ (M+Na$^+$) 415.1363, found 415.1358.

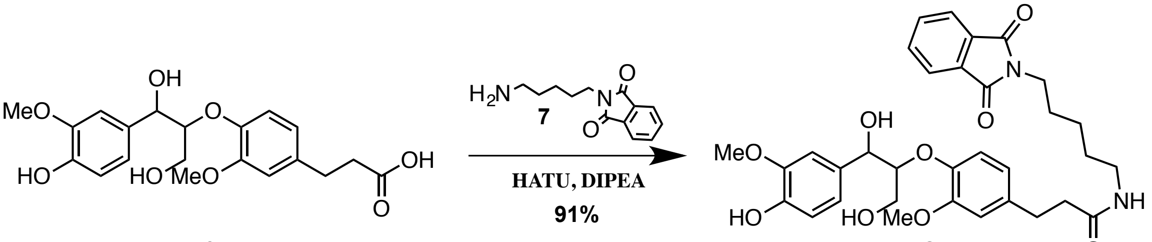

6 8

3-(4-((1,3-dihydroxy-1-(4-hydroxy-3-methoxyphenyl)propan-2-yl)oxy)-3-methoxyphenyl)-N-(5-(1,3-dioxoisoindolin-2-yl)pentyl)propanamideCompound (8)

To a stirred solution of **compound 6** (28 mg, 0.069 mmol) in DMF (2 mL) under nitrogen was added HATU (29 mg, 0.076 mmol) and the resulting mixture was stirred at room temperature for 10 mins. Compound **7** (34 mg, 0.138 mmol) in 1 mL of DMF was added dropwise and the reaction mixture was continued to stir for another 20 mins before the addition of DIPEA (36 uL, 0.207 mmol). The resulting mixture was stirred at room temperature for 12 h. After evaporation of the solvent under reduced pressure, the residue was subjected to column purification to give 40 mg **compound 8** as two diastereomers with ratio of 7:3 in 91% total yield. ^{1}H NMR (600 MHz, $CDCl_3$) δ (ppm) 7.83-7.79 (m, 2H), 7.71-7.67 (m, 2H), 7.03-6.99 (d, 0.7H, *J* = 7.8 Hz), 6.96-6.93 (d, 1H, *J* = 1.2 Hz), 6.90-6.88 (dd, 0.7H, *J* = 7.8, 1.2 Hz), 6.87-6.85 (d, 0.7H, *J* = 7.8Hz), 6.85-6.84 (d, 0.3H, *J* = 2.4 Hz), 6.84-6.82 (d, 0.3H, *J* = 1.8 Hz), 6.81-6.78 (dd, 0.3H, *J* = 8.4, 1.2 Hz), 6.78-6.75 (dd, 1H, *J* = 9.0, 1.8 Hz), 6.72-6.69 (dd, 0.7H, *J* = 7.8, 2.4 Hz), 6.69-6.67 (dd, 0.3H, *J* = 8.4, 1.8 Hz), 5.85-5.75 (br, 1H), 5.62-

5.58(t, 1H, J = 5.4 Hz), 4.94-4.91 (m, 1H), 4.12-4.08 (m, 0.3H), 3.98-3.94 (m, 0.7H), 3.90-3.88 (m, 0.3H), 3.858 (s, 4.2H), 3.852 (s, 0.9H), 3.83 (s, 0.9H), 3.66-3.62 (t, 2H, J = 7.2 Hz), 3.62-3.61 (m, 0.3H), 3.61-3.58 (dd, 0.7H, J = 12.0, 3.0 Hz), 3.48-3.44 (dd, 0.7H, J = 12.6, 4.2 Hz), 3.22-3.16 (m, 2H), 2.95-2.89 (m, 2H), 2.45-2.40 (m, 2H), 1.67-1.62 (m, 2H), 1.57-1.45 (m, 2H), 1.30-1.23 (m, 2H).
^{13}C NMR (150 MHz, $CDCl_3$) Major diastereomer: 172.02, 168.53, 151.02, 148.84, 146.63, 145.95, 145.52, 137.16, 133.94, 132.00, 131.53, 123.18, 121.08, 120.94, 120.17, 114.29, 112.42, 109.43, 89.56, 73.93, 61.06, 55.94, 55.87, 39.28, 38.47, 37.46, 31.44, 28.71, 28.06, 23.80; Minor diastereomer: 172.05, 168.53, 151.31, 146.60, 145.20, 145.06, 137.11, 133.94, 132.00, 131.85, 123.18, 121.03, 120.83, 119.00, 114.21, 112.43, 108.73, 87.37, 72.77, 60.79, 55.94, 55.85, 39.28, 38.47, 37.46, 31.44, 28.71, 28.06, 23.80; HRMS (ESI) *m/z*: Calcd for $C_{33}H_{38}N_2O_9Na$ (M+Na$^+$) 629.2470, found 629.2460.

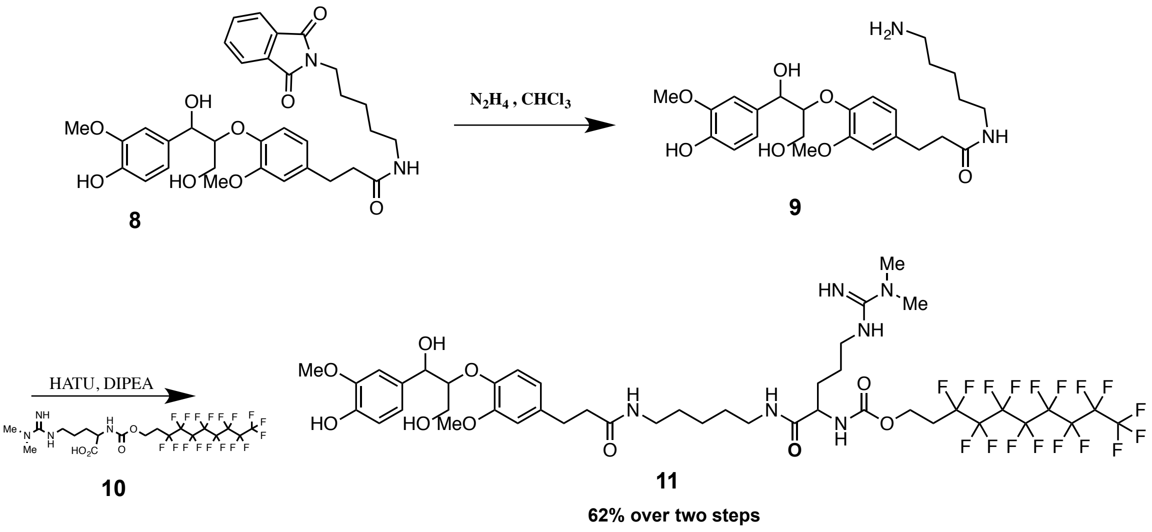

3,3,4,4,5,5,6,6,7,7,8,8,9,9,10,10,10-heptadecafluorodecyl (19-(4-((1,3-dihydroxy-1-(4-hydroxy-3-methoxyphenyl)propan-2-yl)oxy)-3-methoxyphenyl)-3-imino-2-methyl-9,17-dioxo-2,4,10,16-tetraazanonadecan-8-yl)carbamateCompound (11)

To a stirred solution of **compound 8** (64 mg, 0.11 mmol) in $CHCl_3$ (5 mL) was added hydrazine monohydrate (50 μL) and the resulting mixture was stirred at room temperature for 24 h. After evaporation of the solvent under reduced pressure, the residue was pumped under high vacuum to give 48 mg **compound 9**, which was used directly in next week without purification. DEN_B_7 the acid (15 mg, 0.021 mmol) was dissolved in dry DMF (1 mL). With stirring, HATU (9 mg, 0.023 mmol) was added as a solid and the resulting clear solution was stirred for 10 min at room temperature. Compound 10 (20 mg, 0.042 mmol) in 1 mL of dry DMF was injected and the resulting pale yellow solution was stirred for additional 20 min before DIPEA (11 μL, 0.063 mmol) was injected. The resulting yellow solution was stirred at room temperature for 12h. After evaporation of the solvent, the residue was

subjected to column purification to give 15 mg of compound **8** in 62% yield. ^1H NMR (600 MHz, MeOD) δ (ppm) 7.03-7.015 (d, 0.7H, *J* = 1.8 Hz), 7.015-7.00 (d, 0.7H, *J* = 1.2 Hz), 6.97-6.96 (d, 0.3H, *J* = 2.4 Hz), 6.96-6.95 (d, 0.3H, *J* = 1.8 Hz), 6.87-6.84 (m, 2H), 6.84-6.78 (m, 2H), 6.77-6.74 (m, 1H), 6.735-6.715 (m, 1H), 6.715-6.69 (m, 1H), 4.84-4.80 (m, 1H), 4.40-4.30 (m, 2H), 4.25-4.18 (m, 1H), 4.10-4.04 (t, 1H, *J* = 6.6 Hz), 3.852 (s, 0.9H), 3.845 (s, 2.1H), 3.821 (s, 0.9H), 3.817 (s, 2.1H), 3.75-3.68 (m, 2H), 3.48-3.40 (m, 1H), 3.28-3.20 (m, 2H), 3.17-3.06 (m, 2H), 3.48-3.40 (m, 1H), 3.28-3.20 (m, 2H), 3.17-3.06 (m, 4H), 3.01 (s, 6H), 2.88-2.79 (m, 2H), 2.65-2.53 (m, 2H), 1.70-1.52 (m, 5H), 1.50-1.35 (m, 5H), 1.30-1.12 (m, 4H).

^{13}C NMR (150 MHz, MeOD) Major diastereomer: 173.69, 172.766, 156.38, 156.29, 150.43, 150.21, 147.37, 146.45, 146.32, 135.36, 132.38, 120.57, 119.31, 117.99, 112.63, 110.36, 86.07, 72.61, 60.41, 55.09, 55.07, 54.94, 54.65, 48.12, 41.50, 38.85, 38.68, 37.57, 39.09 (2C), 31.13, 29.07, 29.04, 28.58, 28.49 27.05, 24.98; Minor diastereomer: 173.71, 172.774, 156.40, 156.30, 150.39, 150.20, 147.39, 146.49, 146.37, 135.31, 132.74, 120.73, 119.28, 118.02, 112.53, 110.33, 86.12, 72.53, 60.37, 55.09, 55.07, 54.94, 54.65, 48.12, 41.50, 38.85, 38.68, 37.57, 39.09 (2C), 31.13, 29.07, 29.04, 28.58, 28.49 27.05, 24.98; HRMS (ESI) *m/z*: Calcd for $C_{44}H_{56}F_{17}N_6O_{10}$ $(M+H^+)$ 1151.3781, found 1151.3767.

Synthesis of Nonphenolic β-O-4 substrate S18

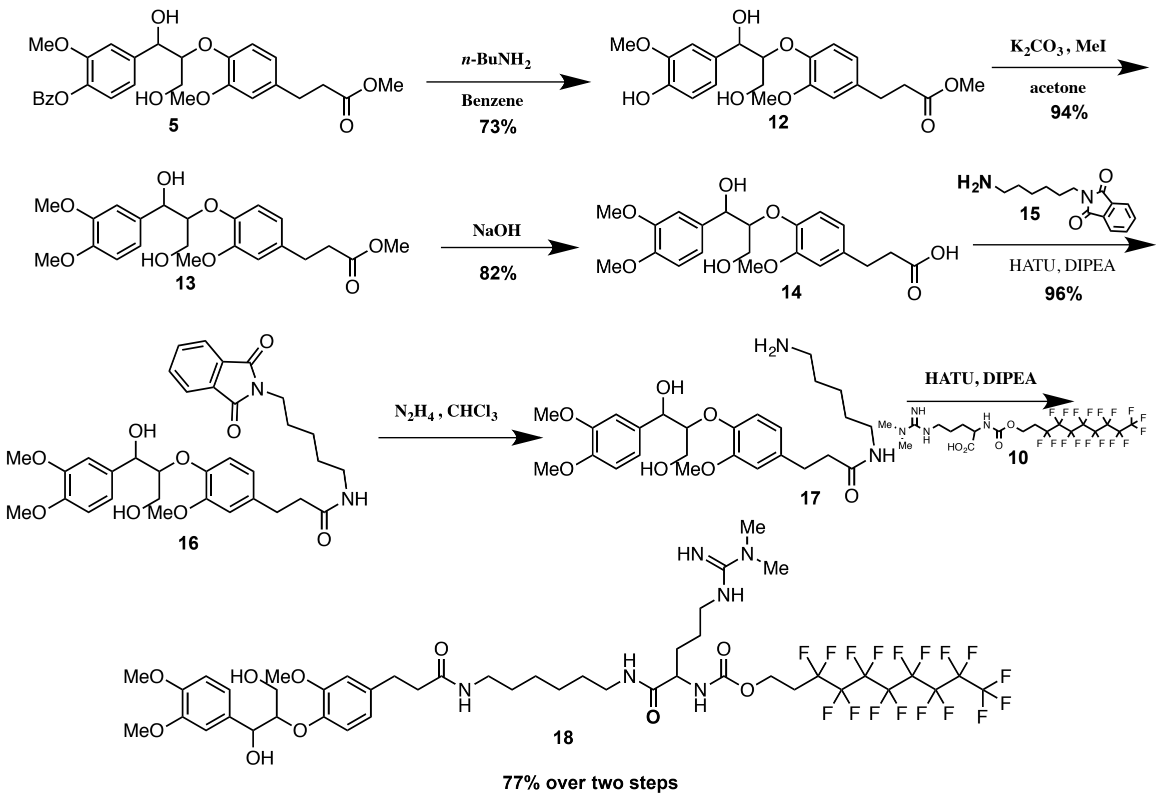

Scheme 2 Synthesis of nonphenolic β-aryl ether substrate 18

Efforts to directly alkylation of the phenol of compound 11 with methyl iodide failed to provide any desired nonphenolic product. Therefore, the synthesis of nonphenolic β-O-4 substrate (Scheme 2) started with the removal of benzoyl group of compound 5 by n-butylamine in benzene (8) followed by methylation with iodomethane in acetone using potassium carbonate as the base (9). Saponification of methyl ester 13 afforded acid 14, which was linked to amine 15 by HATU mediated amide formation. After deprotection of phthalimide, the resulting primary amine was coupled with an acid 10 by HATU mediated amide formation to afford desired nonphenolic β-O-4 substrate 18 with 77% overall yields with two steps.

Detailed Reaction Steps Leading to Compound 18

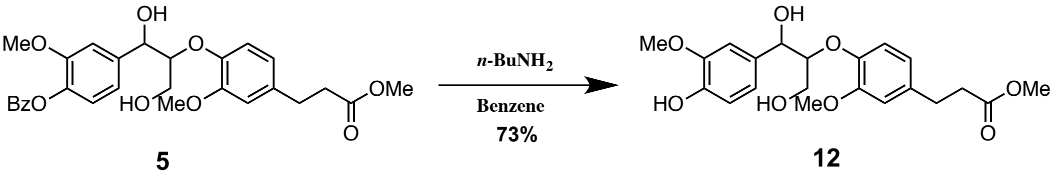

Methyl 3-(4-((1,3-dihydroxy-1-(4-hydroxy-3-methoxyphenyl)propan-2-yl)oxy)-3-methoxyphenyl)propanoate (12)
To a stirred solution of compound **5** (120 mg, 0.24 mmol) in benzene (2 mL) was added *n*-BuNH$_2$ (1.3 mL). The resulting mixture was stirred at room temperature for 24 h. After evaporation of the solvents under reduced pressure and the resulting residue was subjected to column purification to give 70 mg **compound 12** as two diastereomers with ratio of 7:3 in 73% total yield. ^{1}H NMR (600 MHz, CDCl$_3$) δ (ppm) 7.02-6.98 (d, 0.7H, *J* = 8.4 Hz), 6.95-6.90 (m, 1H), 6.88-6.84 (dd, 1H, *J* = 8.4, 1.2 Hz), 6.83 (s, 0.3H), 6.82-6.77 (m, 1H), 6.76-6.73 (d, 0.7H, *J* = 7.8, 1.8 Hz), 6.73-6.69 (m, 1H), 6.69-6.66 (dd, 0.3H, *J* = 7.8, 1.8 Hz), 6.10-5.90 (br, 1H), 4.92-4.88 (m, 1H), 3.99-3.94 (m, 0.7H), 3.90-3.85 (m, 0.3H), 3.84 (s, 2.1H), 3.804 (s, 3H), 3.799 (s, 0.9H), 3.648 (s, 2.1H), 3.644 (s, 2x0.3H), 3.64-3.62 (m, 0.6H), 3.61-3.56 (dd, 0.7H, *J* = 12.6, 3.0 Hz), 3.48-3.41 (dd, 0.7H, *J* = 12.6, 1.8 Hz), 2.92-2.84 (m, 2H), 2.62-2.56 (m, 2H).
^{13}C NMR (150 MHz, CDCl$_3$) Major diastereomer: 173.23, 150.93, 146.71, 146.01, 145.54, 136.56, 131.55, 121.05, 120.70, 120.13, 114.38, 112.30, 109.53, 89.15, 73.88, 61.00, 55.88, 55.81, 51.61, 35.67, 30.60; Minor diastereomer: 173.25, 151.16, 146.64, 145.32, 145.08, 136.43, 132.04, 120.97, 120.48, 119.07, 114.29, 112.32, 108.90, 87.01, 72.77, 60.78, 55.83, 55.81, 51.61, 35.67, 30.60.
HRMS (ESI) *m/z*: Calcd for $C_{21}H_{26}O_8Na$ (M+Na$^+$) 429.1520, found 429.1513.

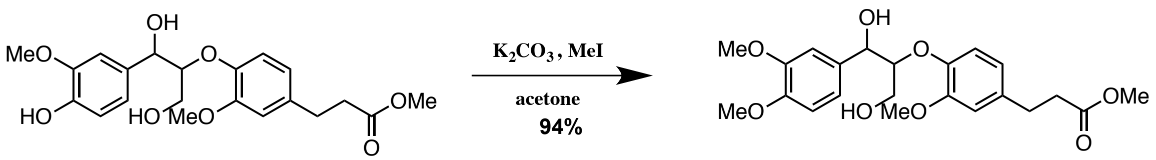

12 **13**

Methyl 3-(4-((1-(3,4-dimethoxyphenyl)-1,3-dihydroxypropan-2-yl)oxy)-3-methoxyphenyl)propanoate (13)

To a stirred solution of **compound 4** (62 mg, 0.15 mmol) in acetone (5 mL) was added methyl iodide (0.1 mL) and K2CO3 (211 mg, 1.53 mmol). The resulting mixture was stirred at room temperature for 12 h. After evaporation of the solvents under reduced pressure, the resulting residue was subjected to column purification to give 60 mg compound **13** as two diastereomers with ratio of 7:3 in 94% total yield. ^{1}H NMR (600 MHz, $CDCl_3$) δ (ppm) 7.06-7.02 (d, 0.7H, *J* = 7.2 Hz), 7.01-6.99 (m, 0.7H), 6.99-6.96 (m, 1H), 6.92-6.89 (d, 0.3H, *J* = 7.2 Hz), 6.87-6.84 (m, 1H), 6.85-6.83 (m, 0.3H), 6.81-6.78 (m, 0.7H), 6.78-6.74 (m, 1H), 6.74-6.71 (m, 0.3H), 5.00-4.96 (m, 1H), 4.15-4.11 (m, 0.3H), 4.02-3.98 (m, 0.7H), 3.93-3.91 (m, 0.3H), 3.90 (s, 2.1H), 3.89 (s, 2.1H), 3.882 (s, 3H), 3.878 (s, 0.9H), 3.869 (s, 0.9H), 3.80-3.76 (m, 0.7H), 3.71-3.69 (m, 0.3H), 3.687 (s, 3H), 3.68-3.64 (m, 0.3H), 3.64-3.60 (m, 0.7H), 3.50-3.44 (m, 0.7H), 2.95-2.90 (m, 2H), 2.65-2.60 (m, 2H). ^{13}C NMR (150 MHz, $CDCl_3$) Major diastereomer: 173.25, 151.04, 149.05, 148.86, 145.95, 136.77, 132.12, 121.15, 120.93, 119.61, 112.30, 110.97, 109.83, 89.44, 73.90, 61.02, 55.90 (2c), 55.87, 51.71, 35.74, 30.68; Minor diastereomer: 173.27, 151.29, 148.97, 148.41, 145.25, 136.66, 132.60, 121.08, 120.75, 118.42, 112.30, 110.96, 109.20, 87.28, 72.68, 60.76, 55.90 (2c), 55.87, 51.71, 35.74, 30.68; HRMS (ESI) *m/z*: Calcd for $C_{22}H_{28}O_8Na$ (M+Na$^+$) 443.1676, found 443.1669.

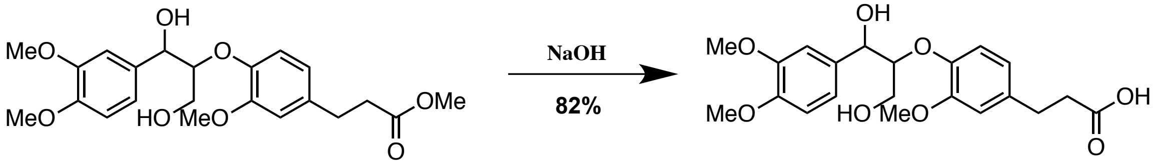

13 **14**

3-(4-((1-(3,4-dimethoxyphenyl)-1,3-dihydroxypropan-2-yl)oxy)-3-methoxyphenyl)propanoic acid (14)

To a stirred solution of **compound 13** (125 mg, 0.30 mmol) in a mixture of methanol (4 mL) and water (2 mL) was added NaOH (1.2 mL, 0.5 M). The resulting mixture was stirred at room temperature for 2 h. After quenching the reaction with HCl (10 mL, 0.1 M), Ethyl acetate was used for extraction. The combined organic layers were concentrated under reduced pressure and the resulting residue was subjected to column purification to give 100 mg compound **14** as two diastereomers with ratio of 7:3 in 82% total yield. ^{1}H NMR (600 MHz, $CDCl_3$) δ (ppm) 7.03-7.00 (d, 0.7H, *J* = 8.4 Hz), 6.98-6.97 (m, 0.7H), 6.97-6.96 (m, 0.7H), 6.95-6.94 (m, 0.3H), 6.90-6.87 (dd, 0.3H, *J* = 7.8, 1.8 Hz), 6.845-6.835 (s, 0.3H), 6.835-6.82 (d, 0.7H, *J* = 1.8 Hz), 6.82-6.81 (s, 0.3H), 6.79-6.77 (d, 0.7H, *J* = 1.8 Hz), 6.77-6.745 (dd, 0.7H, *J* = 7.8, 1.8

Hz), 6.74-6.73 (d, 0.3H, *J* = 2.4 Hz), 6.73-6.70 (dd, 0.3H, *J* = 8.4, 2.4 Hz), 4.98-4.96 (m, 1H), 4.13-4.10 (m, 0.3H), 4.01-3.98 (m, 0.7H), 3.87 (s, 2.1H), 3.864 (s, 2.1H), 3.863 (s, 2.1H), 3.861 (s, 0.9H), 3.858 (s, 0.9H), 3.837 (s, 0.9H), 3.675-3.64 (dd, 0.3H, *J* = 9.0, 3.6 Hz), 3.63-3.60 (dd, 0.7H, *J* = 12.6, 3.0 Hz), 3.48-3.44 (m, 1H), 2.92-2.88 (m, 2H), 2.67-2.62 (m, 2H). ^{13}C NMR (150 MHz, $CDCl_3$) Major diastereomer: 178.10, 150.99, 149.04, 148.86, 145.90, 136.44, 132.03, 121.11, 120.82, 119.56, 112.31, 111.01, 109.90, 89.17, 73.84, 60.95, 55.86, 55.84, 55.82, 35.60, 30.35; Minor diastereomer: 178.10, 151.24, 148.97, 148.43, 145.20, 136.36, 132.48, 121.06, 120.68, 118.43, 112.31, 111.01, 109.26, 87.09, 72.59, 60.62, 55.86, 55.84, 55.82, 35.60, 30.35. HRMS (ESI) *m/z*: Calcd for $C_{21}H_{26}O_8Na$ (M+Na$^+$) 429.1520, found 429.1515.

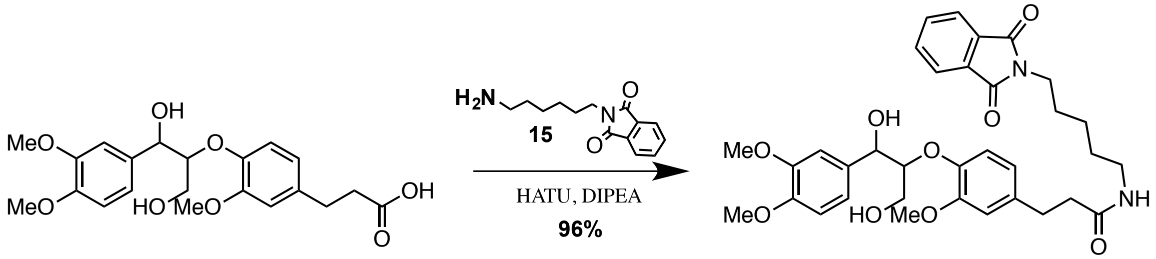

14 **16**

3-(4-((1-(3,4-dimethoxyphenyl)-1,3-dihydroxypropan-2-yl)oxy)-3-methoxyphenyl)-N-(5-(1,3-dioxoisoindolin-2-yl)pentyl)propanamideCompound (16)

To a stirred solution of **compound 14** (47 mg, 0.12 mmol) in DMF (2 mL) under nitrogen was added HATU (50 mg, 0.13 mmol) and the resulting mixture was stirred at room temperature for 10 mins. Compound **15** (56 mg, 0.24 mmol) in 1 mL of DMF was added dropwise and the reaction mixture was continued to stir for another 20 mins before the addition of DIPEA (63 uL, 0.36 mmol). The resulting mixture was stirred at room temperature for 12 h. After evaporation of the solvent under reduced pressure, the residue was subjected to column purification to give 70 mg compound **16** as two diastereomers with ratio of 7:3 in 96% total yield. ^{1}H NMR (600 MHz, $CDCl_3$) δ (ppm) 7.83-7.79 (m, 2H), 7.71-7.67 (m, 2H), 6.97-6.96 (m, 0.7H), 6.96-6.95 (m, 0.7H), 6.95-6.94 (m, 0.3H), 6.88-6.86 (dd, 0.3H, *J* = 8.4, 1.2 Hz), 6.865-6.835 (d, 0.3H, *J* = 8.4 Hz), 6.835-6.815 (d, 0.7H, *J* = 8.4 Hz), 6.82-6.80 (d, 0.3H, *J* = 8.4 Hz), 6.79-6.775 (d, 0.7H, *J* = 1.8 Hz), 6.775-6.76 (d, 0.3H, *J* = 1.8 Hz), 6.74-6.71 (dd, 0.7H, *J* = 8.4, 1.8 Hz), 6.71-6.69 (dd, 0.3H, *J* = 8.4, 1.8 Hz), 5.61-5.56 (t, 1H, *J* = 5.4 Hz), 4.97-4.93 (m, 1H), 4.13-4.09 (m, 0.3H), 3.98-3.94 (m, 0.7H), 3.861 (s, 2.1H), 3.859 (s, 2.1H), 3.855 (s, 3H), 3.849 (s, 0.9H), 3.831 (s, 0.9H), 3.66-3.62 (t, 2H, *J* = 7.8 Hz), 3.62-3.61 (m, 0.3H), 3.61-3.58 (dd, 0.7H, *J* = 12.6, 3.0 Hz), 3.47-3.43 (dd, 0.7H, *J* = 12.6, 3.0 Hz), 3.21-3.15 (m, 2H), 2.95-2.89 (m, 2H), 2.92-2.86 (m, 0.3H), 2.47-2.42 (t, 3H, *J* = 7.8 Hz), 1.67-1.60 (m, 2H), 1.46-1.38 (m, 2H), 1.34-1.26 (m, 4H).

^{13}C NMR (150 MHz, $CDCl_3$) Major diastereomer: 171.96, 168.49, 151.05, 150.23, 149.03, 148.84, 145.90, 137.23, 133.89, 132.51, 132.03, 129.22, 123.14, 121.11, 120.97, 119.56, 112.42, 111.06, 109.93, 89.49, 73.86, 61.0, 55.87, 39.22, 38.49, 37.61, 31.45, 29.22, 28.29, 26.14, 26.00; Minor diastereomer: 171.98, 168.49,

151.35, 150.23, 148.97, 148.40, 145.18, 137.17, 133.89, 132.51, 132.57, 129.22, 123.14, 121.06, 120.89, 118.37, 112.42, 111.06, 109.27, 87.35, 72.69, 60.75, 55.85, 39.22, 38.49, 37.61, 31.45, 29.22, 28.29, 26.14, 26.00;
HRMS (ESI) *m/z*: Calcd for $C_{35}H_{43}N_2O_9$ (M+H$^+$) 635.2963, found 635.2961.

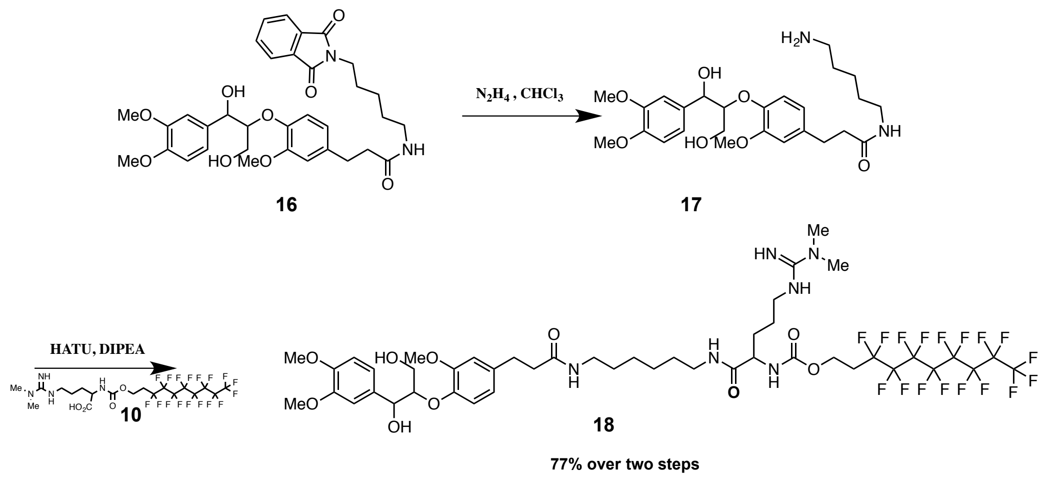

3,3,4,4,5,5,6,6,7,7,8,8,9,9,10,10,10-heptadecafluorodecyl (20-(4-((1-(3,4-dimethoxyphenyl)-1,3-dihydroxypropan-2-yl)oxy)-3-methoxyphenyl)-3-imino-2-methyl-9,18-dioxo-2,4,10,17-tetraazaicosan-8-yl)carbamate Compound (18)

To a stirred solution of compound **16** (28 mg, 0.044 mmol) in $CHCl_3$ (3 mL) was added hydrazine monohydrate (30 μL) and the resulting mixture was stirred at room temperature for 24 h. After evaporation of the solvent under reduced pressure, the residue was pumped under high vacuum to give 22 mg compound **17**, which was used directly in next week without purification. Compound **10** (15 mg, 0.022 mmol) was dissolved in dry DMF (1 mL). With stirring, HATU (9.2 mg, 0.023 mmol) was added as a solid and the resulting clear solution was stirred for 10 min at room temperature. Compound **17** (22 mg, 0.044 mmol) in 1 mL of dry DMF was injected and the resulting pale yellow solution was stirred for additional 20 min before DIPEA (8.6 μL, 0.066 mmol) was injected. The resulting yellow solution was stirred at room temperature for 12h. After evaporation of the solvent, the residue was subjected to column purification to give 20 mg of compound **18** in 77% yield. ^{1}H NMR (600 MHz, MeOD) δ (ppm) 7.08-7.06 (d, 0.7H, *J* = 1.8 Hz), 7.06-7.04 (d, 0.3H, *J* = 1.8 Hz), 6.99-6.97 (dd, 0.7H, *J* = 8.4, 1.2 Hz), 6.96-6.95 (d, 0.7H, *J* = 7.8 Hz), 6.92-6.90 (d, 0.7H, *J* = 8.4 Hz), 6.90-6.87 (d, 0.3H, *J* = 7.8 Hz), 6.86-6.84 (d, 0.7H, *J* = 1.8 Hz), 6.83-6.81 (d, 0.3H, *J* = 7.8 Hz), 6.81-6.79 (d, 0.3H, *J* = 1.8 Hz), 6.72-6.69 (dd, 0.7H, *J* = 8.4, 1.8 Hz), 6.68-6.65 (dd, 0.3H, *J* = 8.4, 1.8 Hz), 4.92-4.90 (m, 1H), 4.43-4.27 (m, 2H), 4.10-4.05 (t, 1H, *J* = 5.4 Hz), 3.847 (s, 2.1H), 3.813 (s, 2.1H), 3.802 (s, 3H), 3.789 (s, 0.9H), 3.782 (s, 0.9H), 3.75-3.71 (m, 1H), 3.47-3.43 (dd, 1H, *J* = 12.0, 5.4 Hz), 3.28-3.22 (m, 2H), 3.19-3.14 (m, 2H), 3.02 (s, 6H), 2.87-2.81 (m, 2H), 2.65-2.53 (m, 2H), 2.47-2.41 (m, 2H), 1.85-1.75 (m, 1H), 1.71-1.55 (m, 3H), 1.52-1.45 (m, 2H), 1.45-1.38 (m, 2H), 1.35-1.20 (m, 6H).^{13}C NMR (150 MHz, MeOD) Major diastereomer: 173.65,

172.71, 156.36, 156.26, 150.21, 148.83, 148.58, 146.38, 135.37, 133.85, 120.51, 119.15, 117.85, 112.50, 111.23, 110.64, 85.80, 72.36, 60.35, 56.73, 56.70, 55.08, 54.99, 54.63, 48.12, 41.49, 38.84, 38.65, 37.56, 37.13 (2C), 31.13, 29.06, 28.81, 28.77, 25.93, 25.92, 24.99; Minor diastereomer: 173.66, 172.72, 156.36, 156.26, 150.43, 148.74, 148.47, 146.02, 135.32, 134.19, 120.36, 119.42, 118.04, 112.58, 111.14, 110.75, 84.96, 72.49, 60.61, 56.77, 56.67, 55.11, 55.04, 54.63, 48.12, 41.49, 38.84, 38.65, 37.56, 37.13 (2C), 31.13, 29.06, 28.81, 28.77, 25.93, 25.92, 24.99; HRMS (ESI) *m/z*: Calcd for $C_{46}H_{60}F_{17}N_6O_{10}$ ($M+H^+$) 1179.4094, found 1179.4073.

References:

(1) Zea, S.; Pepijn, P.; Frits Van Der, K.; Stefania, T.; Gadi, R. “Lignin solubilisation and gentle fractionation in liquid ammonia” *Green Chemistry*, **2015**, 17, 325-334.
(2) Weinstein, D. A.; Gold, M. H. *Holzforschung*, **1979**, *33*, 134-135.
(3) Wu, A.; Patrick, B. O.; Chung, E.; James, B.R. “Hydrogenolysis of β-O-4 lignin model dimers by aruthernium-xantphos catalyst” *Dalton Transactions*, **2012**, *41(36)*, 11093-11106.
(4) Kawai, S.; Okita, K.; Sugishita, K.; Tanaka, A.; Ohashi, H. “Simple method for synthesizing phenolic β-O-4 dilignols” *J. Wood Sci.* **1999**, *45*, 440-443.
(5) Xing, X.; Padmanaban, D.; Yeh, L-A.; Cuny, G.D. “Utilization of a copper-catalyzed diaryl ether synthesis for the preparation of berbenachalcone” *Tetrahedron* **2002**, *58*, 7903-7910.
(6) Ding, X.; Li, Y.; Zhou, M.; Han, L.; Zhang, Z.; Ba, Q.; Li, J.; Wang, H.; Liu, H.; Wang, R. “De Novo Design, Synthesis and Evaluation of Benzylpiperazine Derivatives as highly Selective Binders of Mcl-1” *ChemMedChem* **2013**, *8*, 1986-2014.
(7) Deng, K.; Takasuka, T.E.; Heins, R.; Cheng, X.; Bergeman, L.F.; Shi, J.; Aschenbrener, R.; Deutsch, S.; Singh, S.; Sale, K.L; Simmons, B.A.; Adams, P.A.; Singh, A.K.; Fox, B.G.; Northen, T.R. “Rapid Kinetic Characterization of Glycosyl Hydrolases Based on Oxime Derivatizatin and Nanostructure-Initiator Mass Spectrometry (NIMS)” *ACS Chemcial Biology* **2014**, *9*, 1470-1479.
(8) Bell, K. H. “facile selective aminolysis of phenolic benzoates with 1-butanamine in benzene” *Tetrahedron Lett* **1986**, *27*, 2263-2264.
(9) Yue, F.; Lu, F.; Ralph, S.; Ralph, J. “Identification of 4-O-5 units in softwood lignins via definitive lignin models and NMR” *Biomacromolecules* **2016**, *17*, 1909-1920.

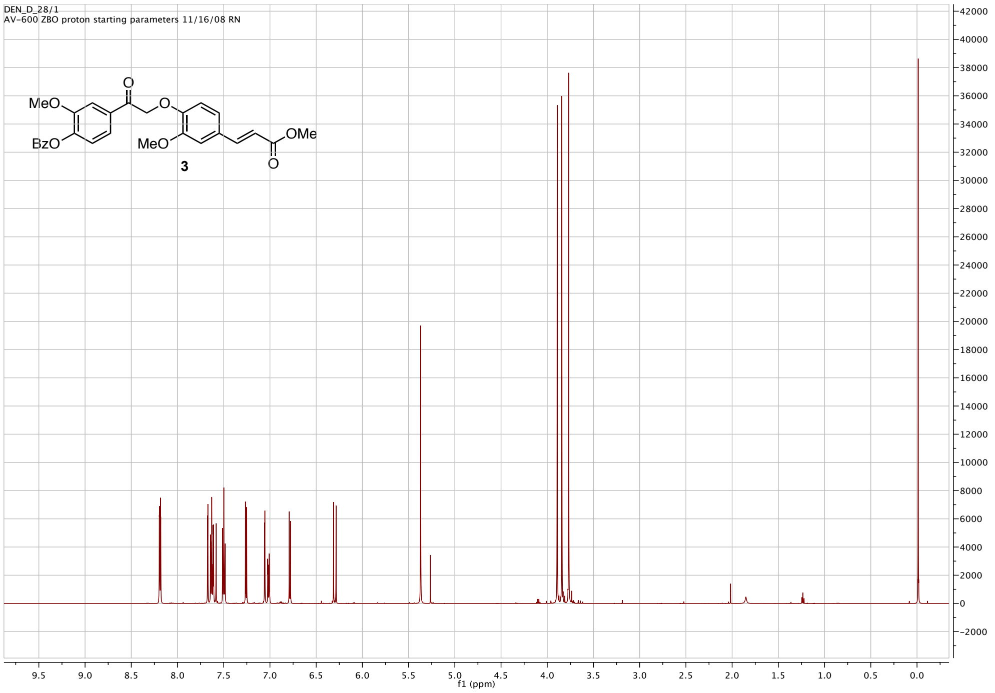

Figure 1 Proton NMR spectra for compound 3

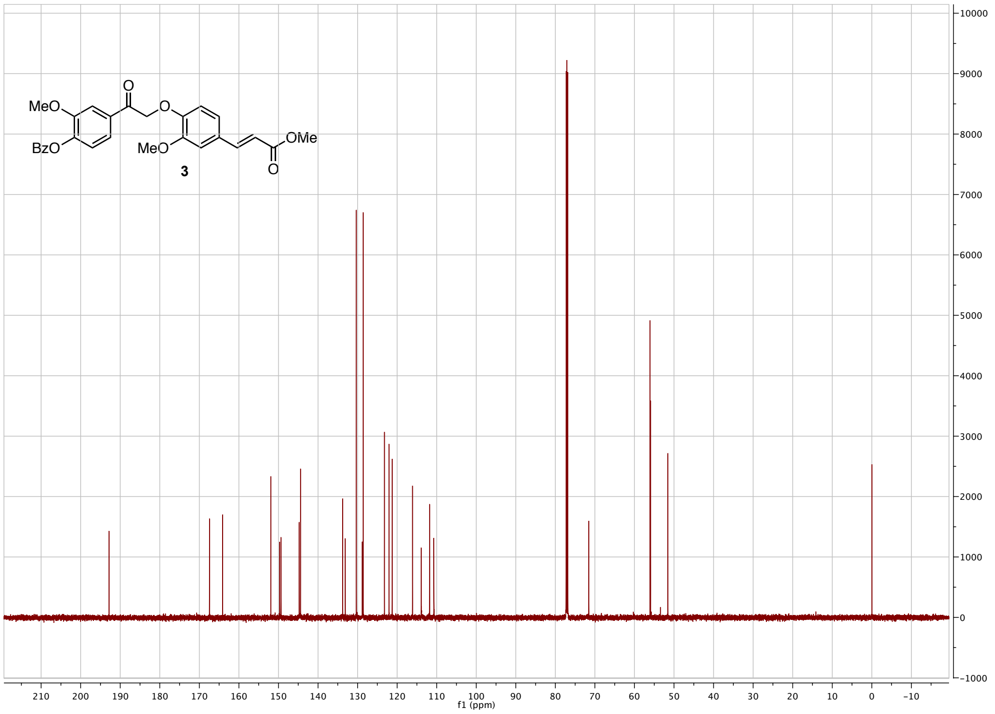

Figure 2 ^{13}C NMR spectra of compound 3

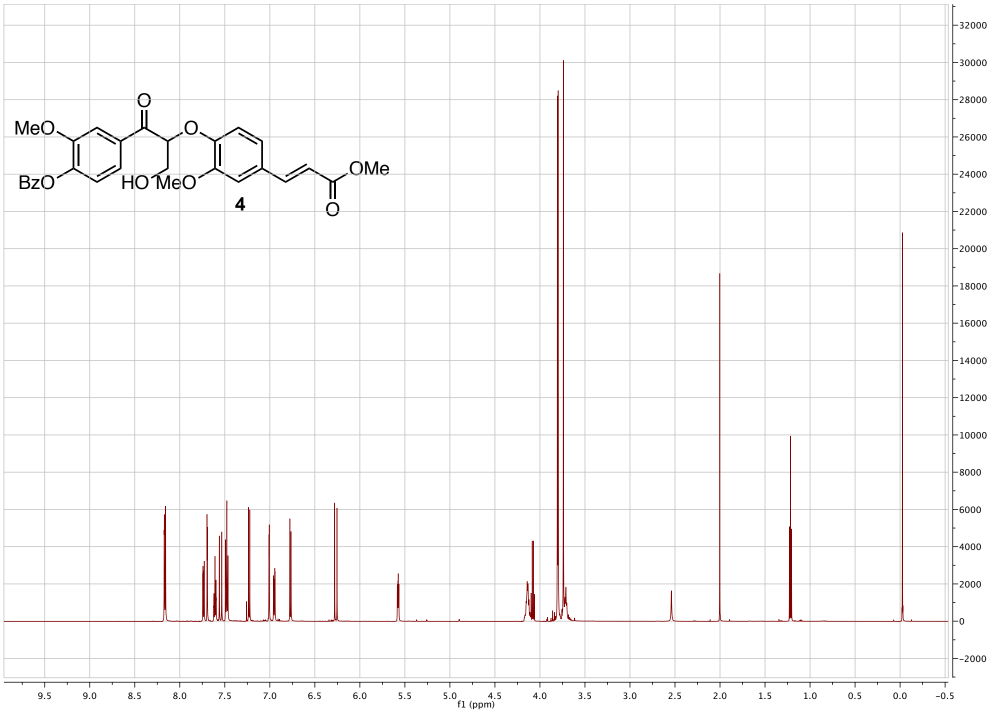

Figure 3 Proton NMR spectra of compound 4

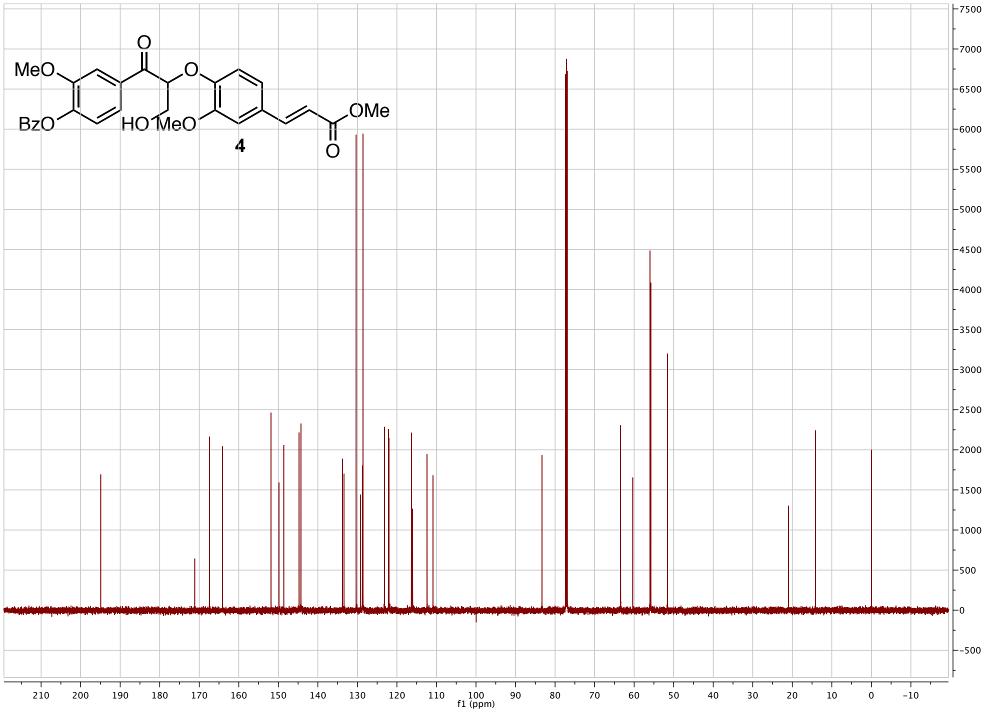

Figure 4 ^{13}C NMR spectra of compound 4

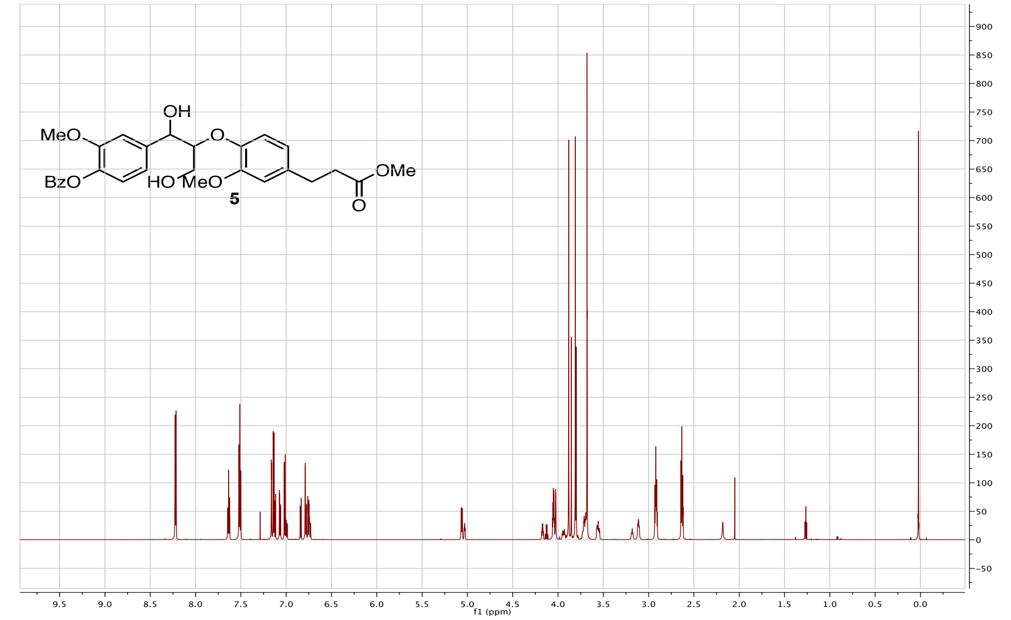

Figure 5 Proton NMR spectra of compound 5

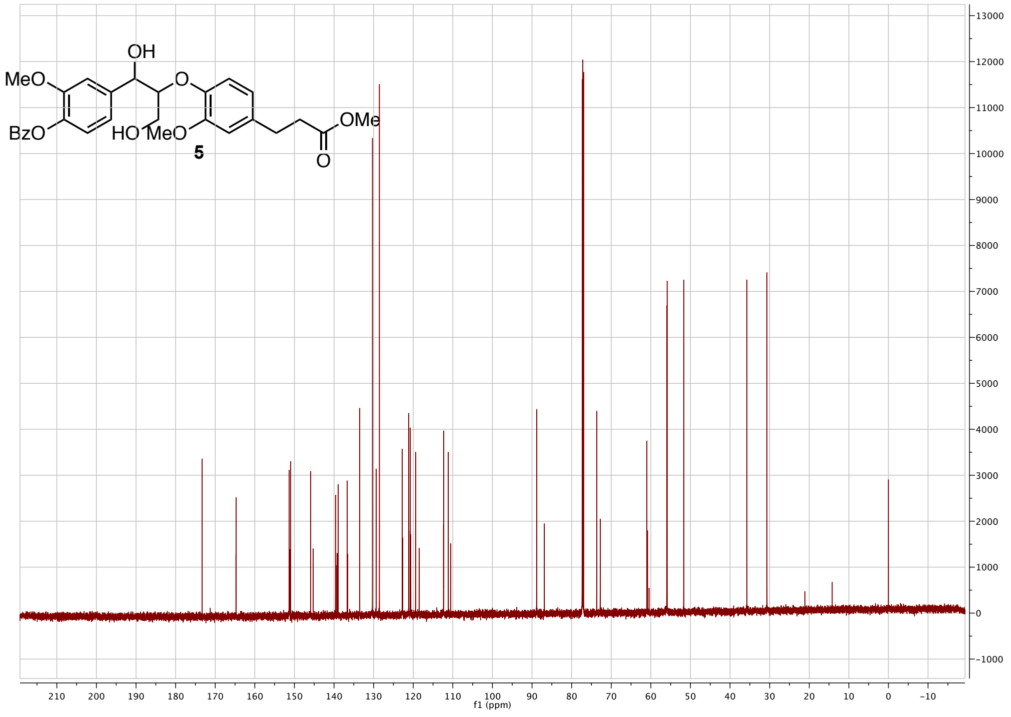

Figure 6 ^{13}C NMR spectra of compound 5

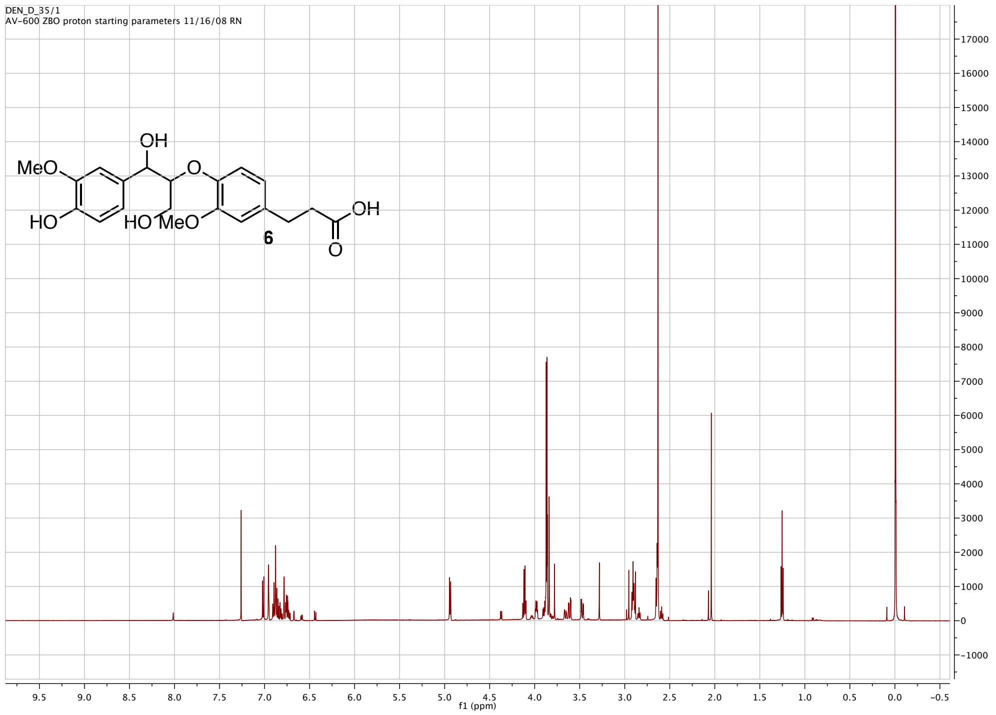

Figure 7 Proton NMR spectra of compound 6

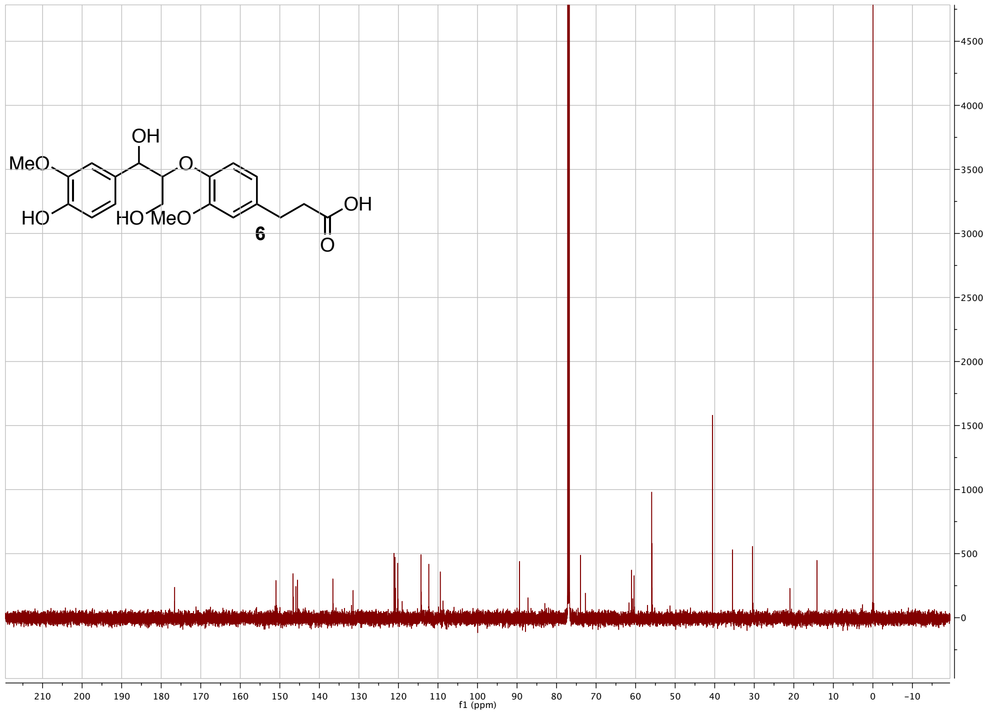

Figure 8 13C NMR spectra of compound 6

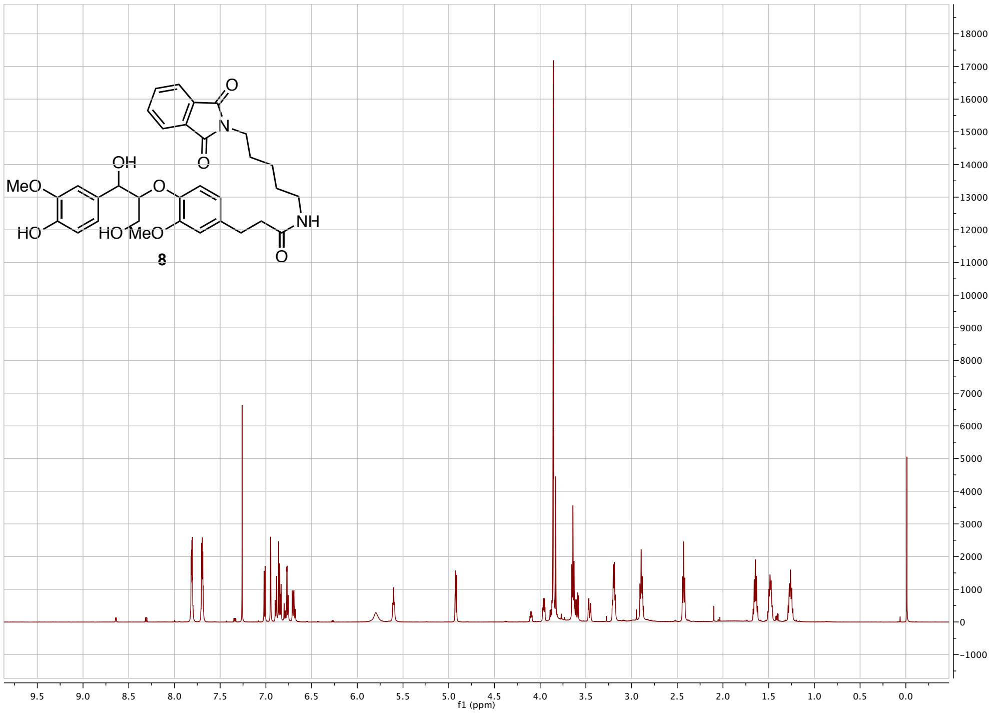

Figure 9 Proton NMR spectra of compound 8

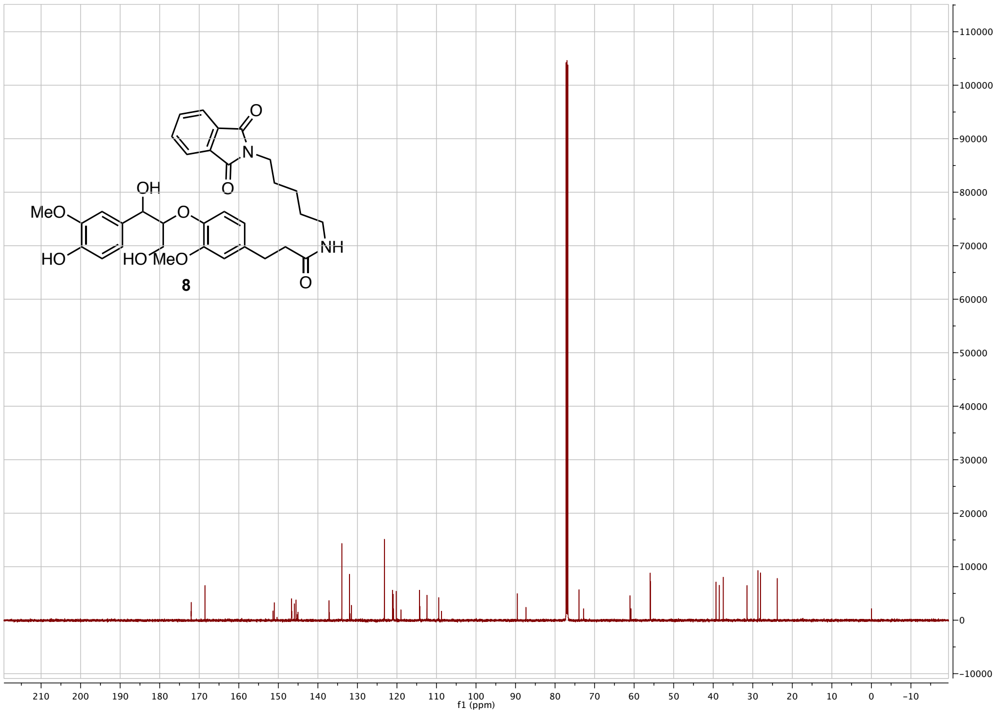

Figure 10 ^{13}C NMR spectra of compound 8

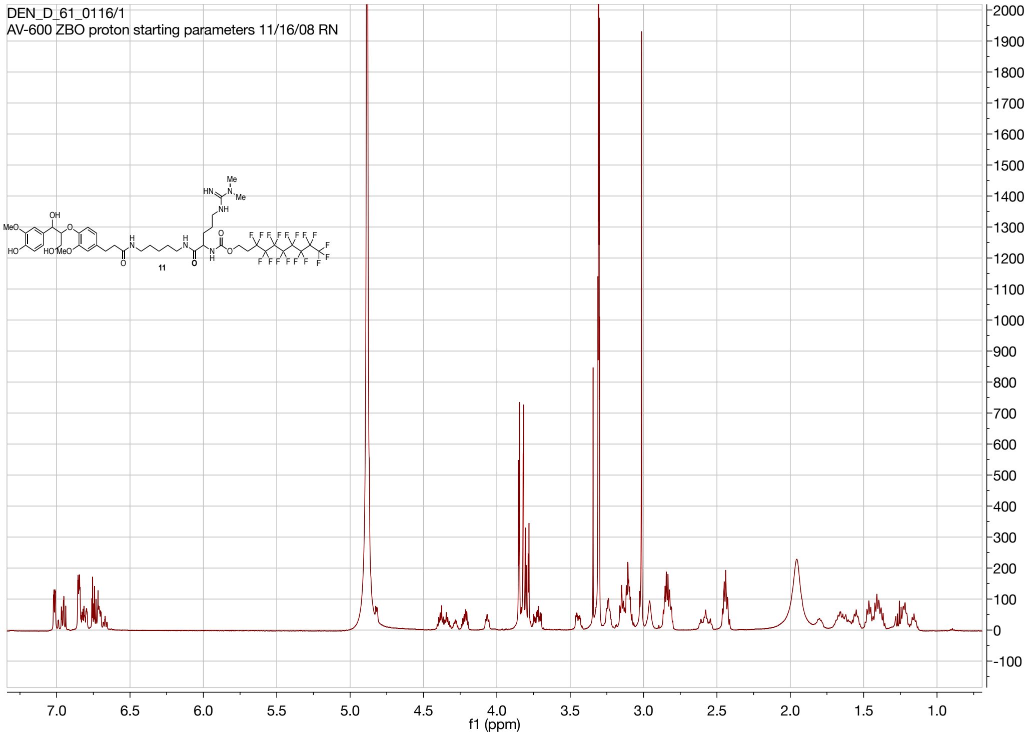

Figure 11 Proton NMR spectra of compound 11

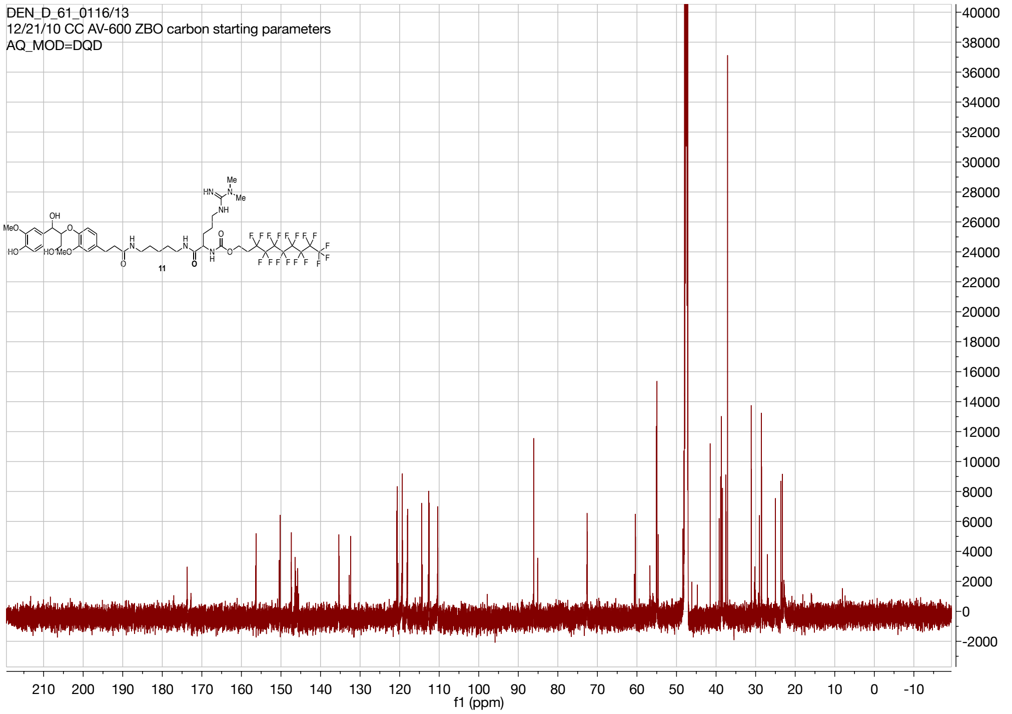

Figure 12 ^{13}C NMR spectra of compound 11

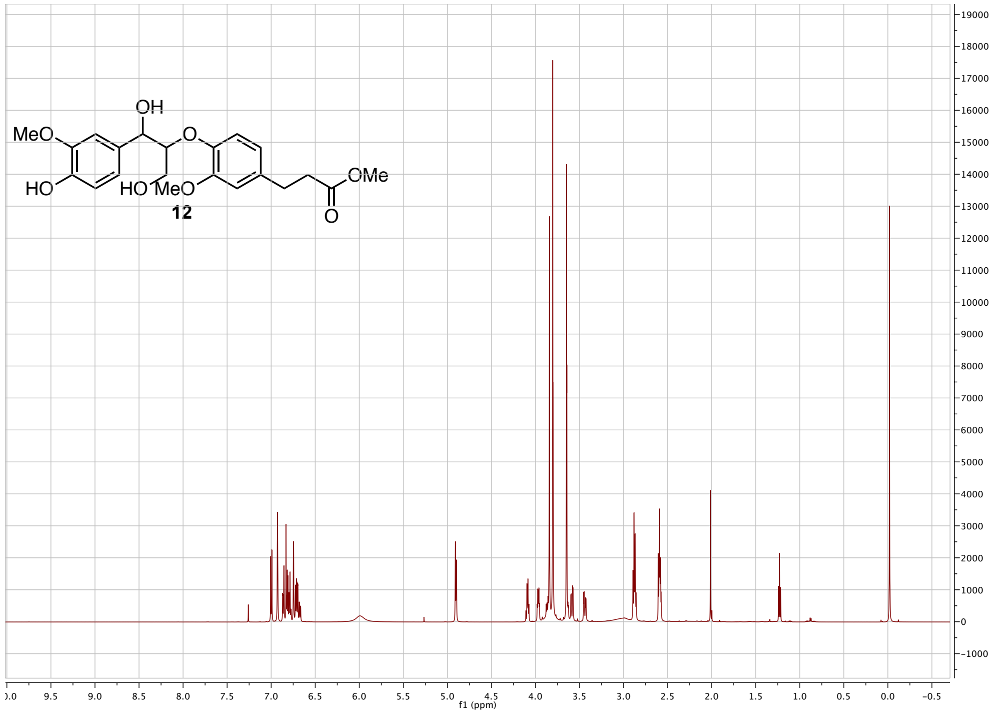

Figure 13 Proton NMR spectra of compound 12

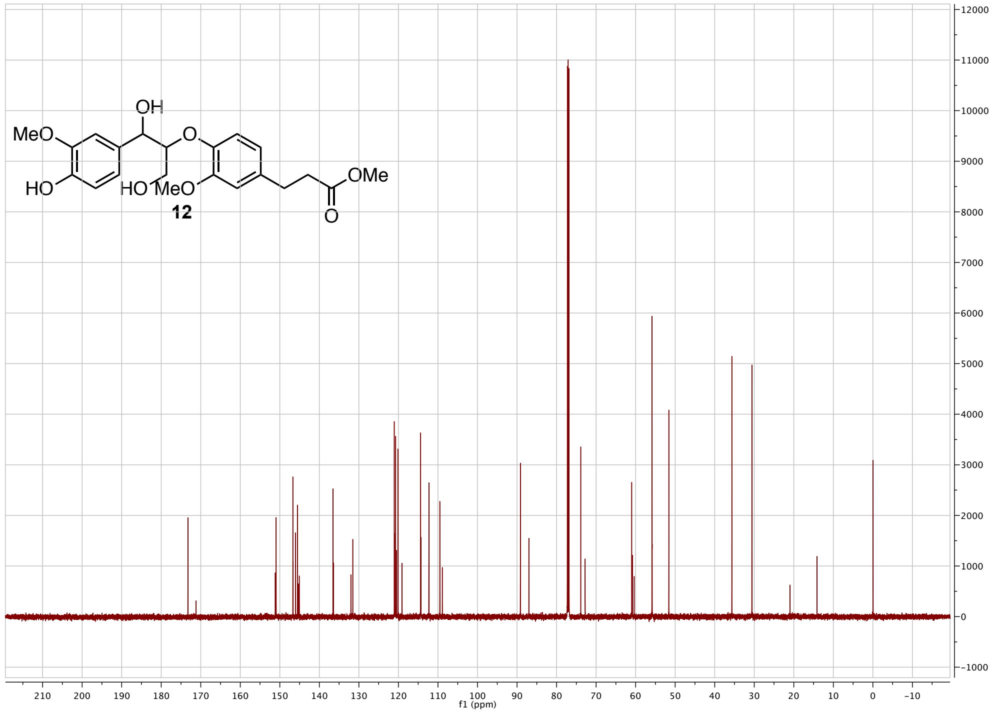

Figure 14 ¹³C NMR spectra of compound 12

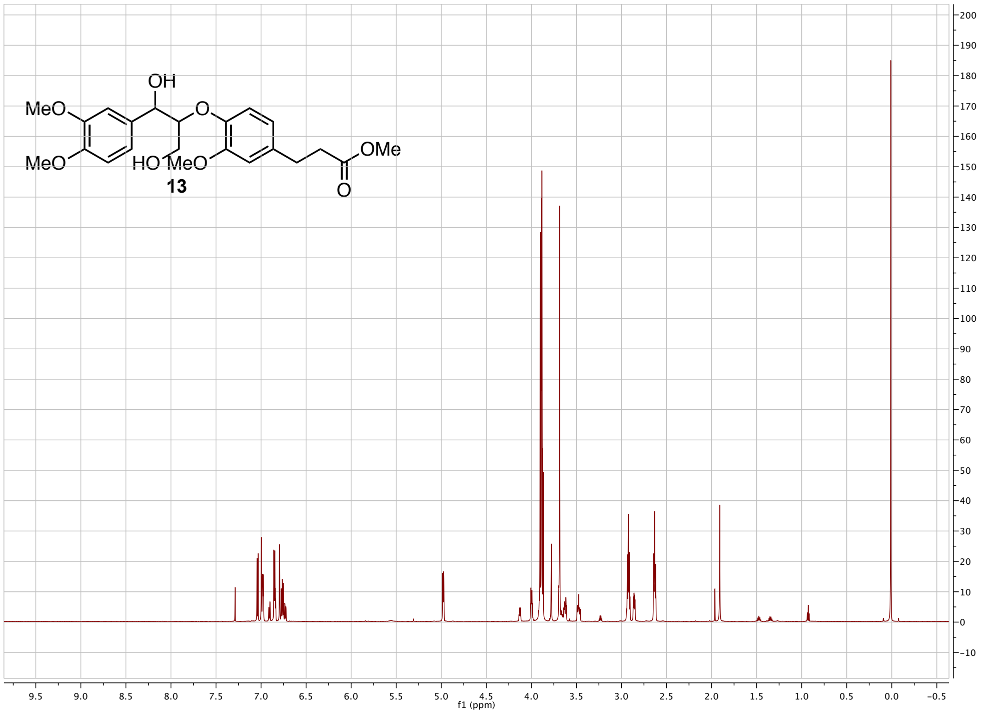

Figure 15 Proton NMR spectra of compound 13

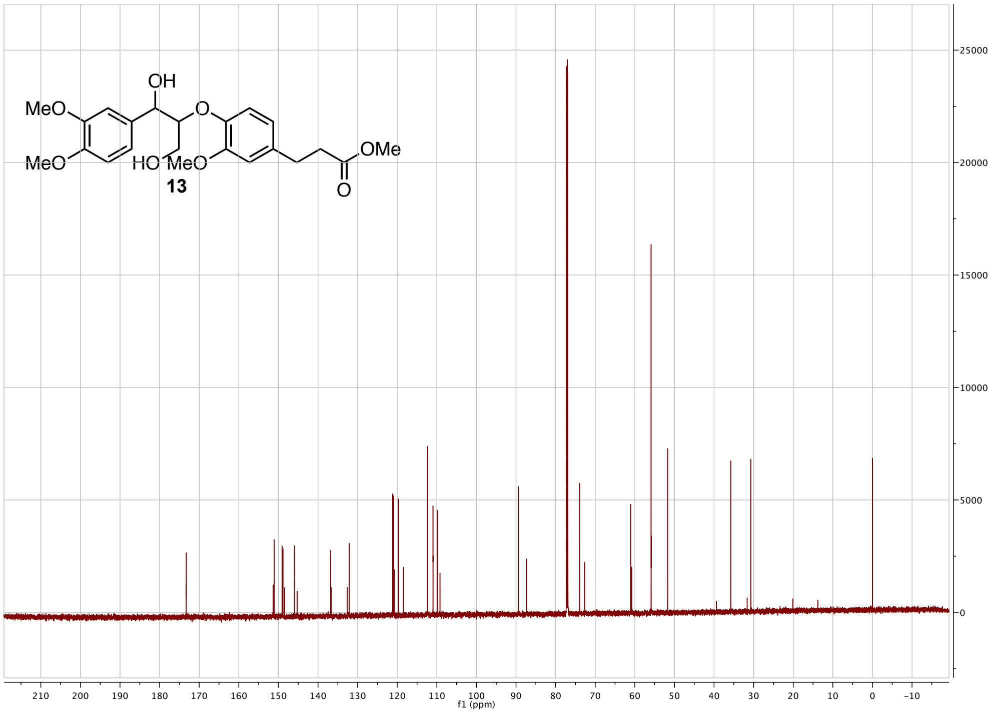

Figure 16 ^{13}C NMR spectra of compound 13

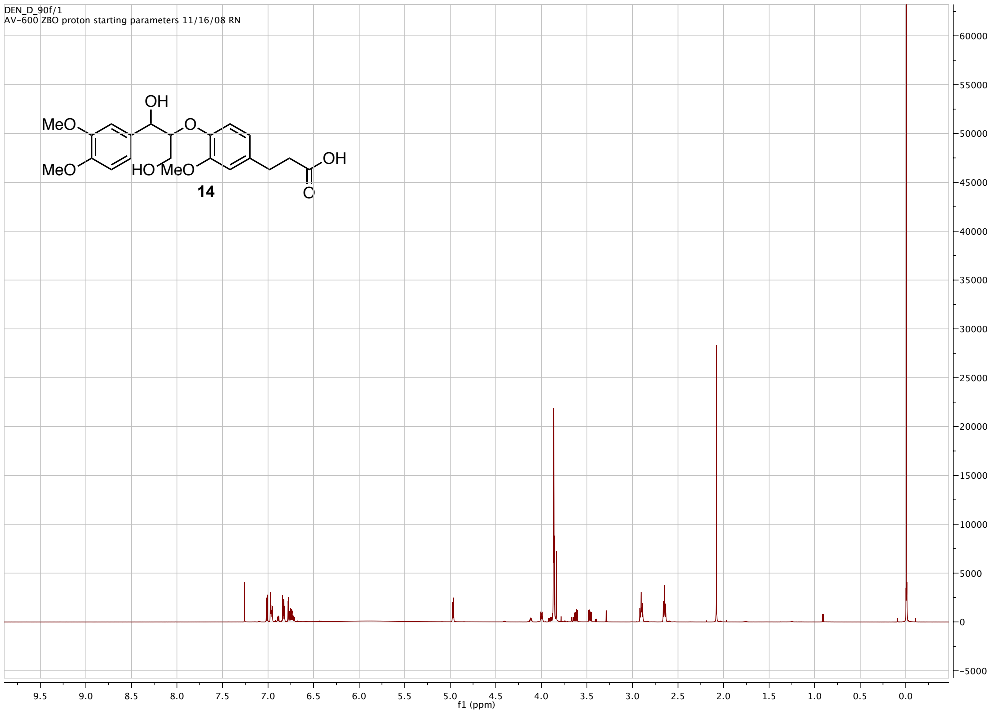

Figure 17 Proton NMR spectra of compound 14

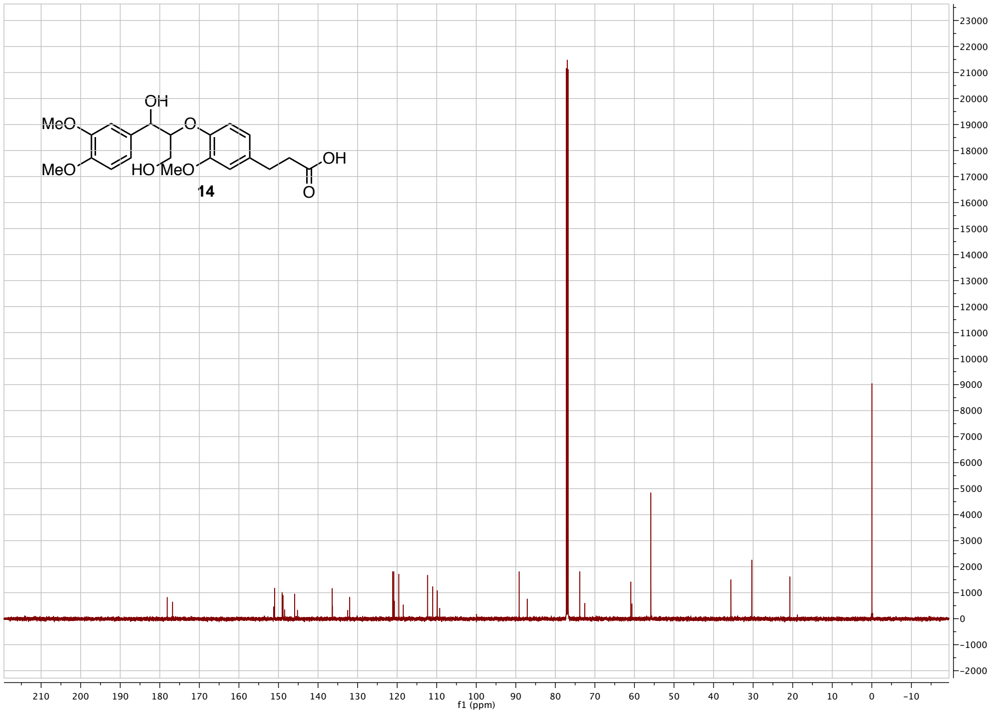

Figure 18 ^{13}C NMR spectra of compound 14

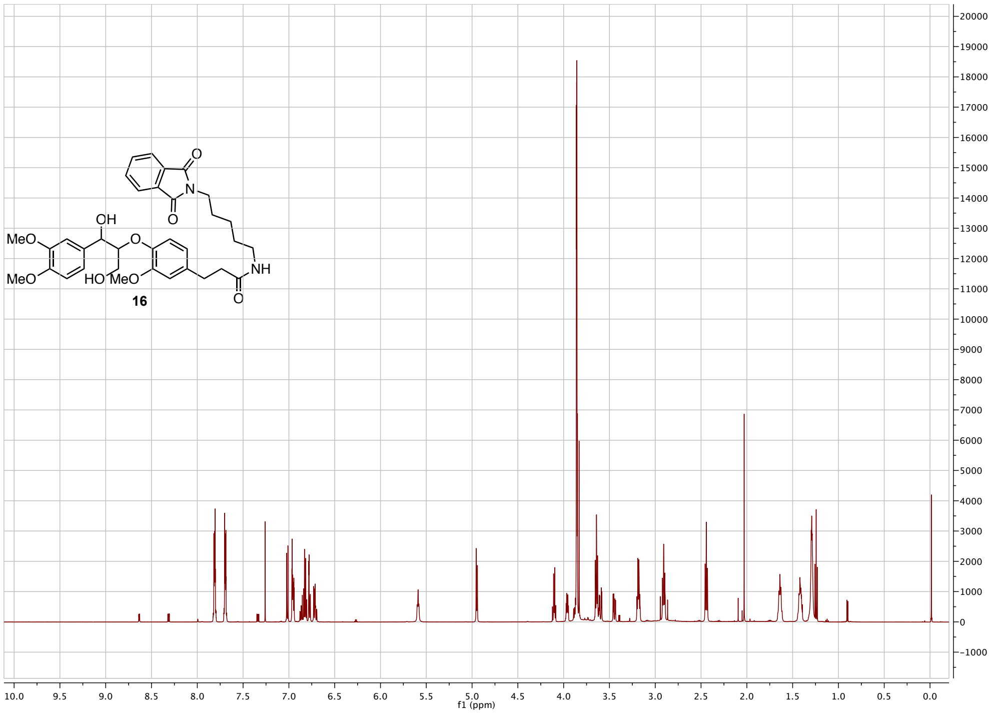

Figure 19 Proton NMR spectra of compound 16

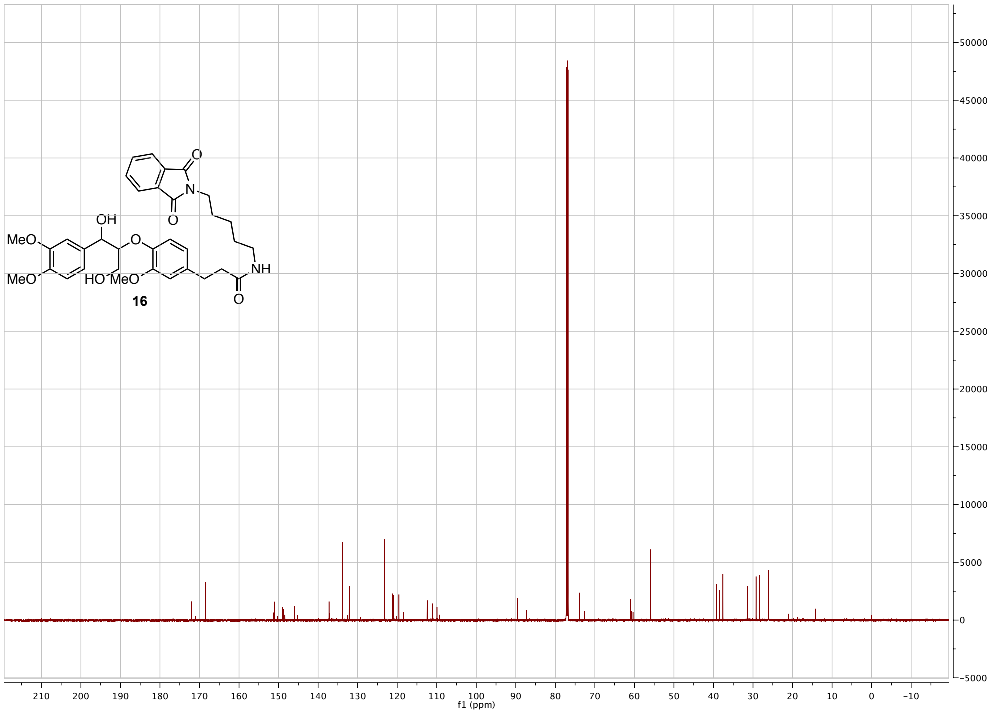

Figure 20 ^{13}C NMR spectra of compound 16

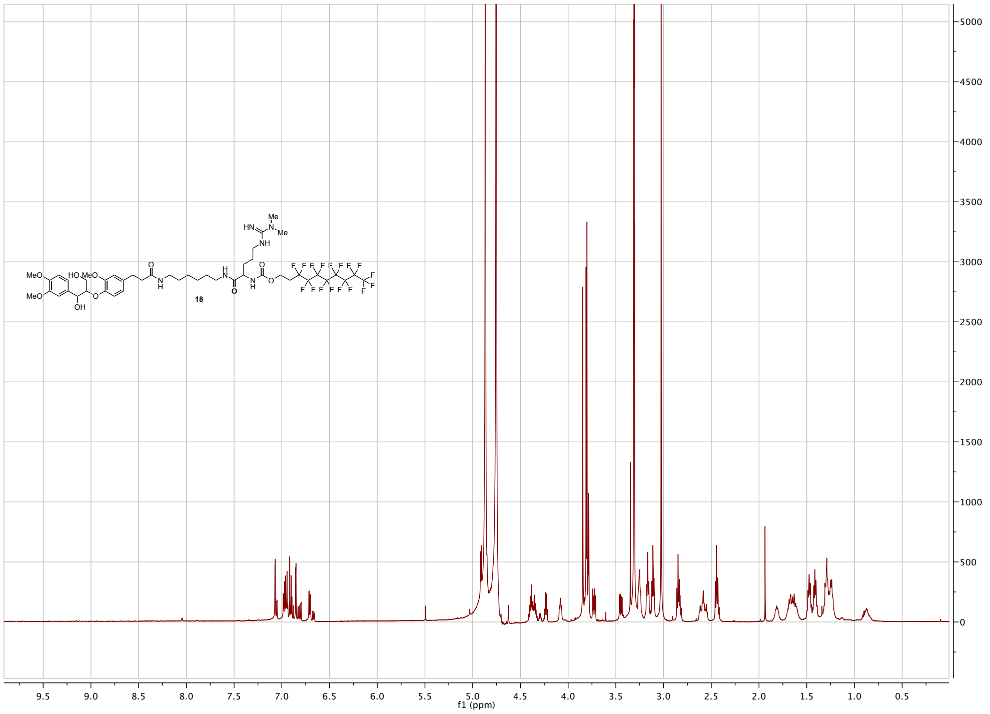

Figure 21 Proton NMR spectra of compound 18

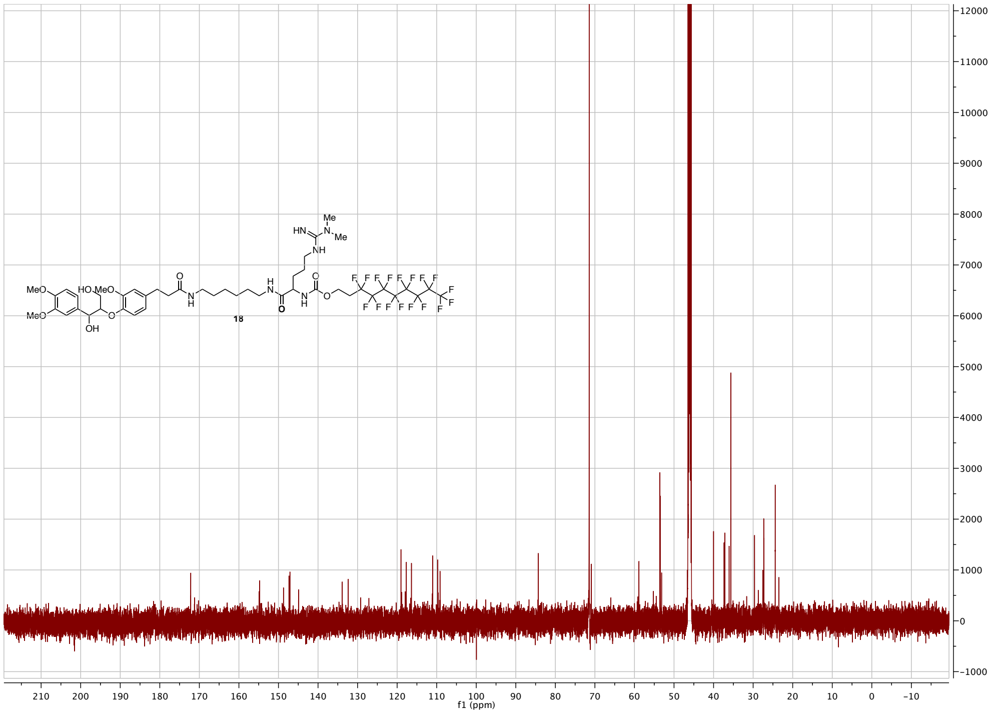

Figure 22 ^{13}C NMR spectra of compound 18
